# Unsymmetrical naphthalene diimides as electrochromic materials: tailoring structure for controlled optical and redox properties

**DOI:** 10.1039/d6ra08357a

**Published:** 2026-10-06

**Authors:** Marcin Nowacki, Sergiusz Napierała, Monika Wałęsa-Chorab

**Affiliations:** a Faculty of Chemistry, Adam Mickiewicz University in Poznań Uniwersytetu Poznańskiego 8 61-614 Poznań Poland mchorab@amu.edu.pl

## Abstract

Naphthalene diimides (NDIs) are attractive electron-deficient building blocks for electrochromic materials owing to their excellent electrochemical stability and reversible redox behavior. Herein, we report three unsymmetrical NDI derivatives bearing aromatic *N*-substituents with progressively increasing electron-donating ability, enabling systematic tuning of their electrochemical and optical properties. All compounds exhibit two reversible reduction processes, whereas donor-functionalized derivatives additionally undergo reversible oxidation, resulting in multistate electrochromism. Spectroelectrochemical studies reveal three, four, and five distinct optical states for NDI-1, NDI-2, and NDI-3, respectively, accompanied by pronounced absorption changes across the visible and near-infrared regions. Owing to its broad electrochemical window and rich optical response, NDI-3 was employed as the active material in a solid-state electrochromic device, which demonstrated reversible multicolor switching, optical modulation in the visible and near-infrared regions, switching times of 1.4–8.7 s, with particularly good stability observed for the radical anion/dication transition. These findings demonstrate that molecular engineering of unsymmetrical NDI derivatives provides an effective strategy for developing multistate electrochromic materials with tunable optical properties for advanced electrochromic devices and energy-efficient smart window applications.

## Abstract 2

Naphthalene diimides (NDIs) are attractive electron-deficient building blocks for electrochromic materials owing to their excellent electrochemical stability and reversible redox behavior.

## Graphical abstract

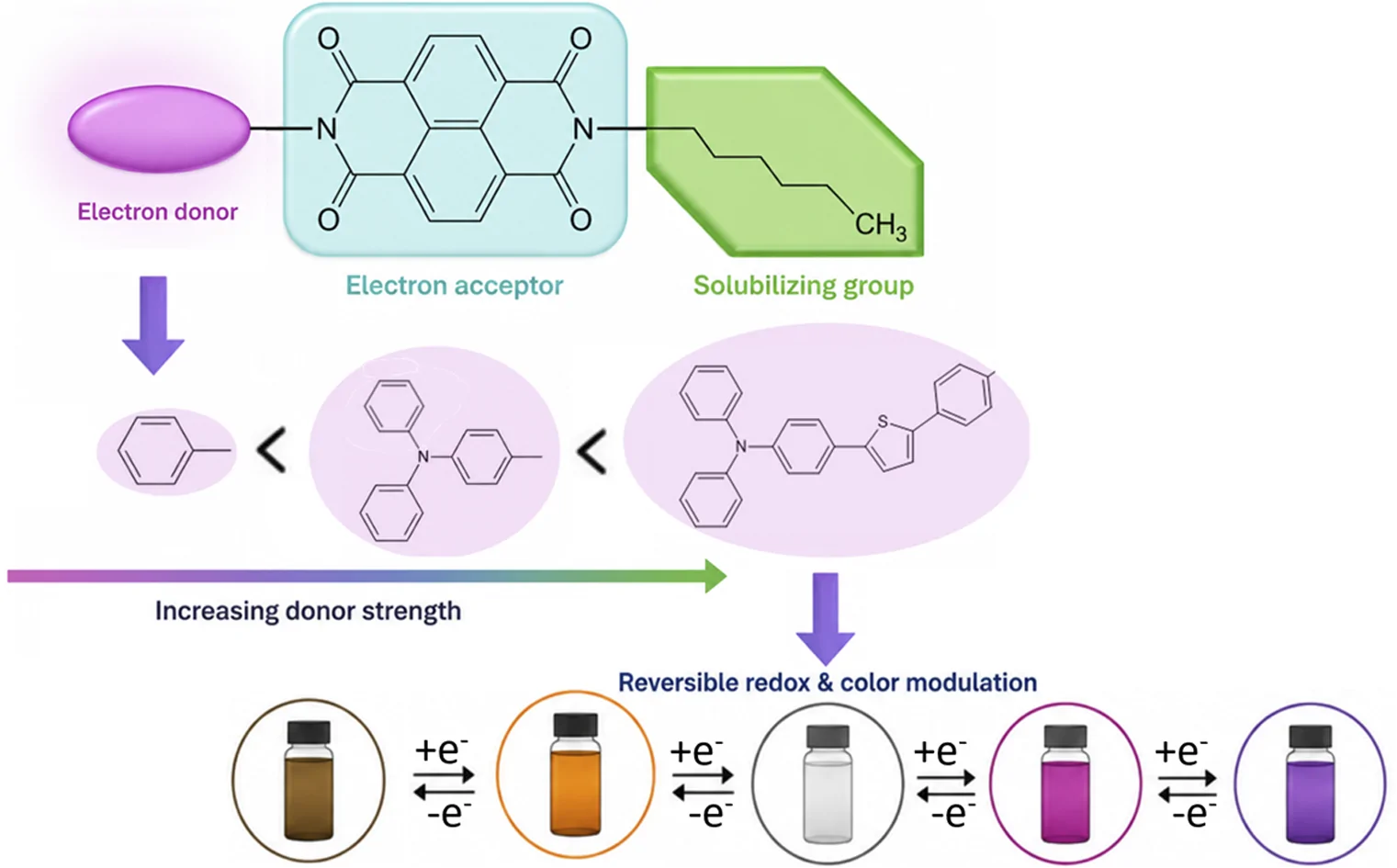

## Introduction

Naphthalene diimides (NDIs) represent a versatile class of π-conjugated molecules characterized by electron-deficient aromatic cores. Owing to their strong electron-accepting character, efficient electron-transport properties, and remarkable resilience to thermal and oxidative degradation, NDIs have emerged as key components in the design of organic electronic materials.^1–5^ Their utility spans applications such as photovoltaic systems and optoelectronic devices.^6^

Beyond their established importance in organic electronics, NDIs have attracted growing attention due to their structural versatility and wide-ranging functional properties. Their strong tendency to form stable supramolecular assemblies has positioned them as valuable components in supramolecular chemistry, particularly in the design of host–guest systems^7,8^ and mechanically interlocked molecules such as catenanes and rotaxanes.^9–12^ In addition, NDIs have shown considerable promise in diverse fields, including ion-channel mimics,^13^ catalytic systems,^14^ biomedical applications,^15^ and as non-fullerene acceptors in next-generation photovoltaic devices.^16–18^ This broad applicability highlights the significance of NDIs as multifunctional organic materials worthy of continued investigation.

Aromatic diimides, particularly NDIs, are widely recognized as effective electron acceptors in donor–acceptor systems due to their strong electron-withdrawing character. Their planar, π-conjugated frameworks promote efficient π–π stacking interactions with electron-rich donor species, which, in certain cases, can give rise to materials with intriguing luminescence properties.^19,20^ These non-covalent interactions support the formation of charge-transfer (CT) complexes, thereby enhancing charge separation and transport.^21,22^ Moreover, they enable fine-tuning of the optical and electronic properties of the resulting materials, underscoring the critical role of NDIs in the design of functional organic systems.

NDI-based compounds typically undergo two reversible one-electron reduction processes, corresponding to the [NDI]/[NDI]˙^−^ and [NDI]˙^−^/[NDI]^2−^ redox couples. These well-defined electrochemical transitions are accompanied by distinct and visually observable color changes, resulting from the formation of the radical anion and dianion species. Such behavior makes NDIs particularly attractive for applications in electrochromic devices, where controlled color modulation is essential.^23,24^ One of the key advantages of NDIs lies in their synthetic accessibility. Their core structure can be readily obtained *via* condensation of naphthalene dianhydride with a variety of primary amines, allowing systematic control over electronic and steric parameters. Their functionalization by *N*-substitution of alkyl chains leads to enhancement of solubility and processability in organic solvents, while core substitution can dramatically change the photophysical properties.^25^

Molecular donor–acceptor engineering provides a rational approach to address this challenge. The electron-donating strength of a substituent can modify the frontier molecular orbitals, particularly the HOMO, while the electron-deficient NDI core can retain its characteristic reduction behavior. Increasing the donor strength may therefore facilitate reversible oxidation and introduce additional redox states without eliminating the two characteristic NDI-centered reductions. Such orthogonal control of reduction and oxidation processes could provide a direct molecular route toward increasing the number of accessible optical states. Although donor–acceptor interactions have been widely exploited to tune the electronic and photophysical properties of π-conjugated materials,^26–28^ a systematic relationship between the donor strength of NDI *N*-substituents, the resulting redox-state landscape, and multistate electrochromic response has not been fully established. Unsymmetrical NDI derivatives offer an attractive molecular architecture for this purpose. The two imide substituents can be differentiated, allowing the NDI acceptor core to be combined with an electronically tunable aromatic donor on one side while retaining a structurally distinct substituent on the other. This design provides an opportunity to modulate donor–acceptor electronic coupling and the oxidation potential of the molecule while preserving the intrinsic reduction processes of the NDI core. A systematic increase in the electron-donating character and π-conjugation of the aromatic *N*-substituent may consequently provide a means of progressively expanding the accessible electrochemical and optical-state space.

Despite the considerable progress in the development of NDI-based electrochromic materials, most reported molecular systems have focused primarily on exploiting the intrinsic two-step reduction of the NDI core or on introducing additional electroactive units through core functionalization and extended π-conjugation. In this context, the role of the imide *N*-substituent as a deliberate tool for controlling multielectrochromic behavior remains comparatively less explored. Systematic studies correlating the electron-donating character and π-conjugation of aromatic *N*-substituents with the accessible redox states and corresponding optical responses are scarce.

This work presents three structurally related unsymmetrical NDI derivatives (NDI-1–NDI-3, Fig. 1), featuring progressively more electron-donating and π-conjugated aromatic *N*-substituents.

**Fig. 1:**
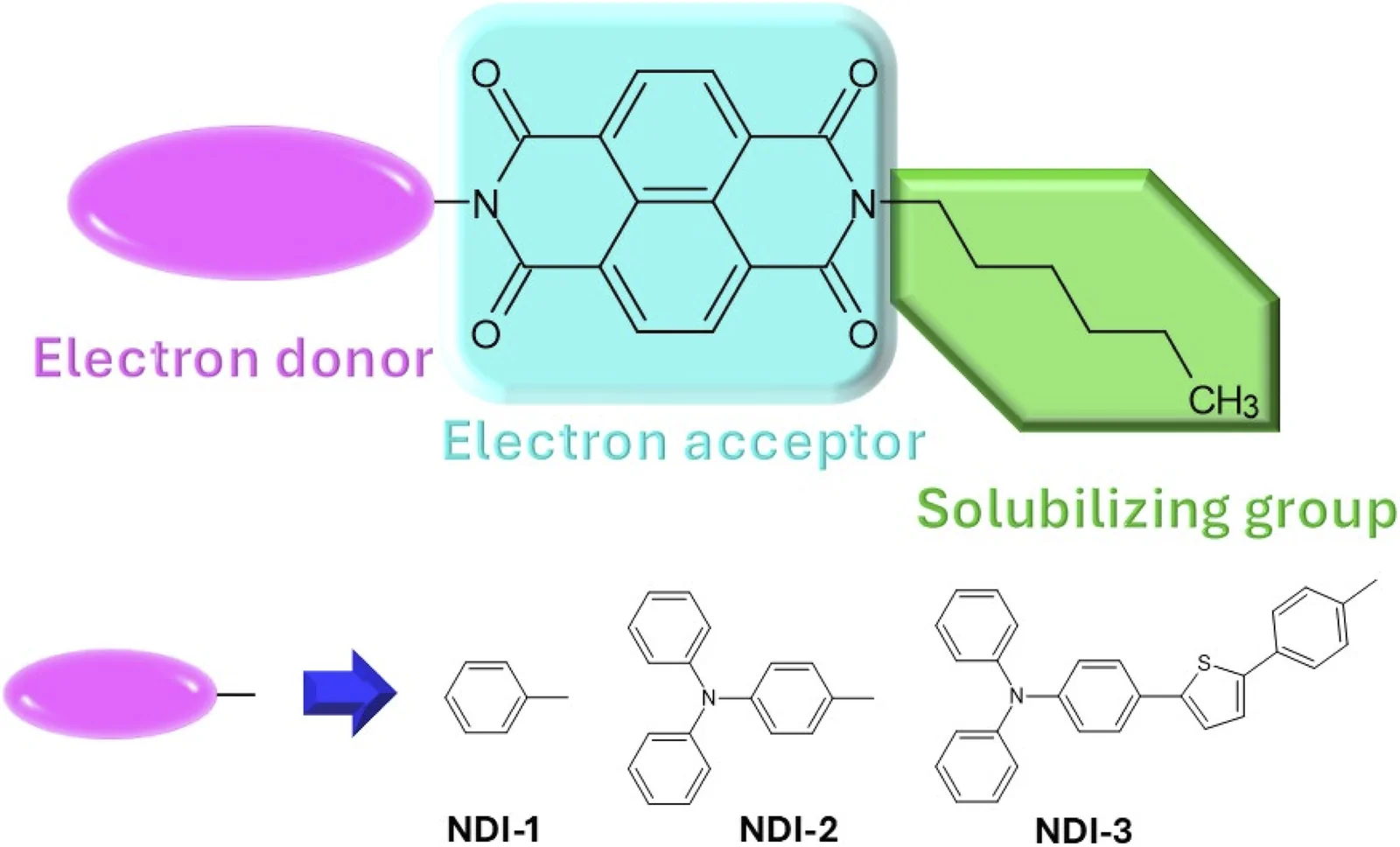
The schematic representation of NDI-1–NDI-3.

The substituent-dependent changes were examined to determine their influence on the electrochemical and optical properties of the NDI derivatives and on the number of accessible redox and electrochromic states. Comprehensive electrochemical and spectroelectrochemical studies were performed for all three compounds, while the electrochromic device performance was further demonstrated using NDI-3 as the most redox-rich derivative.

## Results and discussion

### Materials preparation

The target compounds were synthesized *via* a multistep procedure, as outlined in Fig. 2 and S1.

**Fig. 2:**
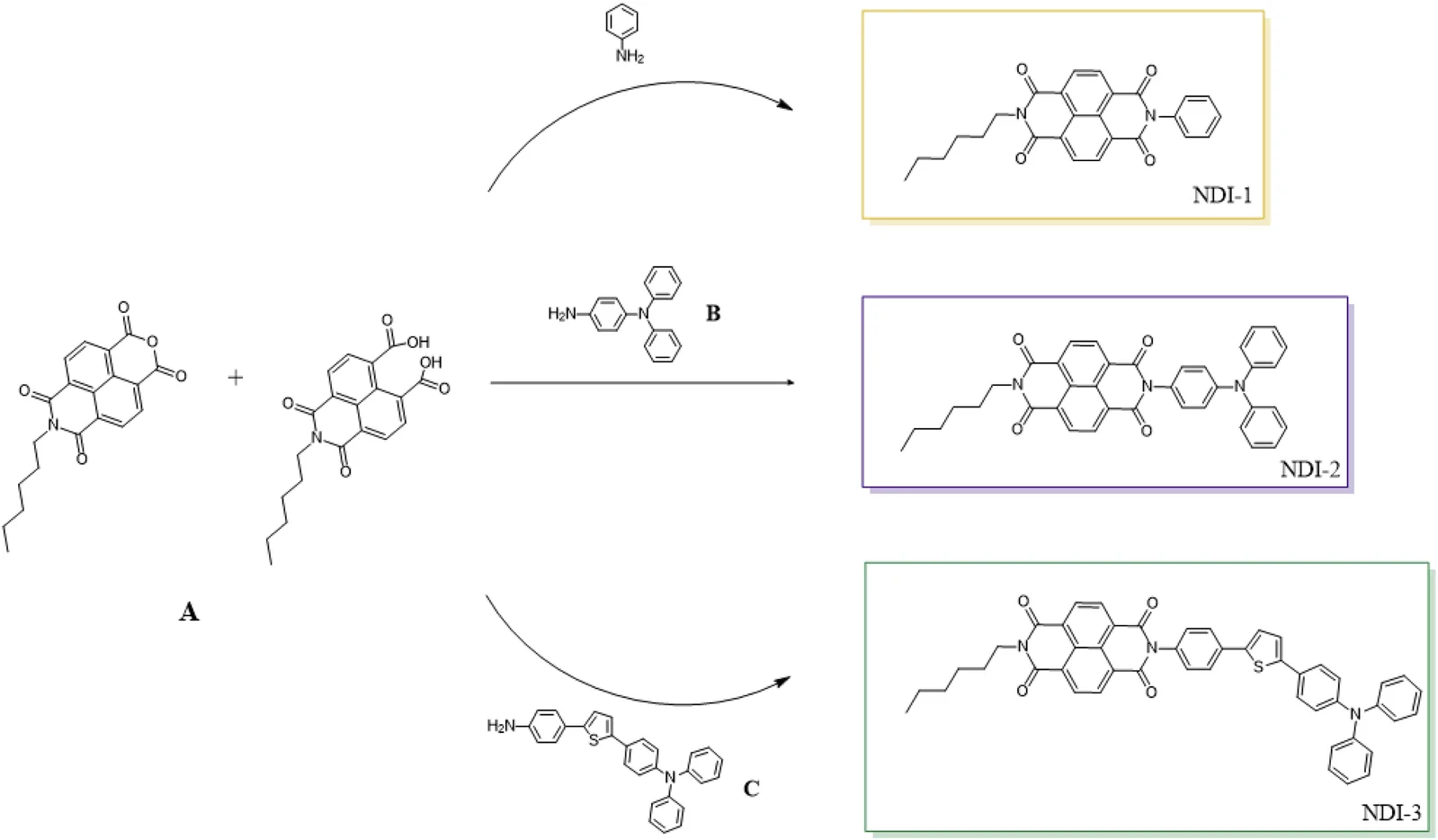
Synthetic scheme of NDI-1–NDI-3.

The first step involved the preparation of a naphthalenemonoimide bearing an *N*-substituted hexyl chain (Fig. S1). As previously reported by Pantos *et al.*, a one-step reaction of 1,4,5,8-naphthalenetetracarboxylic dianhydride (NDA) with one equivalent of amine typically yields a statistical mixture of dianhydride, monoimide, and diimide in a 1 : 2 : 1 ratio.^29^ To circumvent this limitation, a modified two-step protocol, as proposed by Pantos *et al.*, was employed.^29^ Compound A was obtained *via* a microwave-assisted reaction, in which a 1 : 1 molar mixture of NDA and hexylamine was first heated at 60 °C for 40 minutes, followed by an increase in temperature to 140 °C for an additional 15 minutes. The initial low-temperature step promotes selective nucleophilic attack on NDA rather than NMI, thus enhancing reaction specificity. This protocol enables the efficient preparation of NMIs with high yields and facilitates straightforward purification. Compound A was obtained in over 90% yield and was readily purified by precipitation from an aqueous 1 M hydrochloric acid solution. The aromatic region of the NMR spectrum of A (Fig. S4) exhibits a multiplet at 8.67 ppm and two doublets at 8.54 ppm and 8.18 ppm. The signal at 8.67 ppm was assigned to aromatic protons of the naphthalene core in the closed form, featuring a cyclic monoanhydride structure. In contrast, the two doublets correspond to the open form, in which the anhydride group is hydrolyzed to two carboxylic acid functionalities.^29^ The presence of the latter is further supported by a broad singlet observed at 13.57 ppm, characteristic of carboxylic acid protons. These data confirm that A was obtained as a mixture of closed and open forms, with a molar ratio of 1 : 2 as determined from the NMR spectral integration. The obtained material was subsequently subjected to a second condensation reaction with the appropriate aromatic amine to synthesize unsymmetrical naphthalene diimides (NDI-1–NDI-3). A series of aromatic amines with different electron-donating strengths were selected for the reaction. The amine components employed included aniline, 4′-aminotriphenylamine (B),^30^ and 4-(5-(4-aminophenyl)thiophen-2-yl)-*N*,*N*-diphenylaniline (C). This synthetic strategy enabled the preparation of materials with well-defined optical and electrochemical characteristics. The condensation reactions were performed in a microwave reactor at 180 °C for 1.5 h in *N*,*N*-dimethylformamide (DMF) as the solvent. Crude products were precipitated by addition to a 1 M aqueous HCl solution and subsequently purified by chromatographic techniques. The chemical structure and purity of the target compounds were confirmed by spectroscopic methods (Fig. S5–S16).

### Electrochemical properties

The electrochemical properties of NDI-1–NDI-3 were investigated by cyclic voltammetry (CV) in 0.1 M solution of Bu_4_NClO_4_ in propylene carbonate (PC) as the supporting electrolyte at a scan rate of 100 mV s^−1^. Measurements were carried out using a 3 mm-diameter platinum working electrode, a non-aqueous Ag/Ag^+^ reference electrode, and a platinum wire serving as the counter electrode. Ferrocene was used as an external standard, and all potentials were referenced to the ferrocene/ferrocenium (Fc/Fc^+^) redox couple.

It was found that the number and potentials of redox processes in NDI derivatives strongly depend on the structure of the aromatic *N*-substituent (Fig. 3 and Table 1). All compounds exhibit two reversible reduction waves, corresponding to the formation of the radical anion and dianion species on the NDI core. Enhanced π-conjugation within the aromatic *N*-substituent results in a clear shift of the reduction potentials toward less negative values, from −1.14 V (NDI-1) to −1.08 V (NDI-3) for the neutral/radical-anion couple, and from −1.62 V to −1.50 V for the radical-anion/dianion couple. As is well known, the electronic coupling of the NDI radical anion is not confined to the diimide core but also extends to the *N*-substituents.^3^ The observed shift of the NDI core reduction potentials from NDI-1 to NDI-3 can be attributed to electronic interactions between the diimide core and the π-conjugated *N*-substituents. Charge delocalization provided by the aromatic substituents adjacent to the imide nitrogen likely lowers the local charge density at the reduction site, thereby facilitating the first reduction and stabilizing the resulting radical anion.^31^ The reverse electron-transfer processes from the dianionic to the radical-anionic state were detected at −1.49 V for NDI-1 and NDI-2, and −1.50 V for NDI-3. Furthermore, the subsequent oxidation of the radical anion to the neutral NDI species was observed at −1.0 V, −0.99 V, and −0.97 V for NDI-1 through NDI-3.

**Fig. 3:**
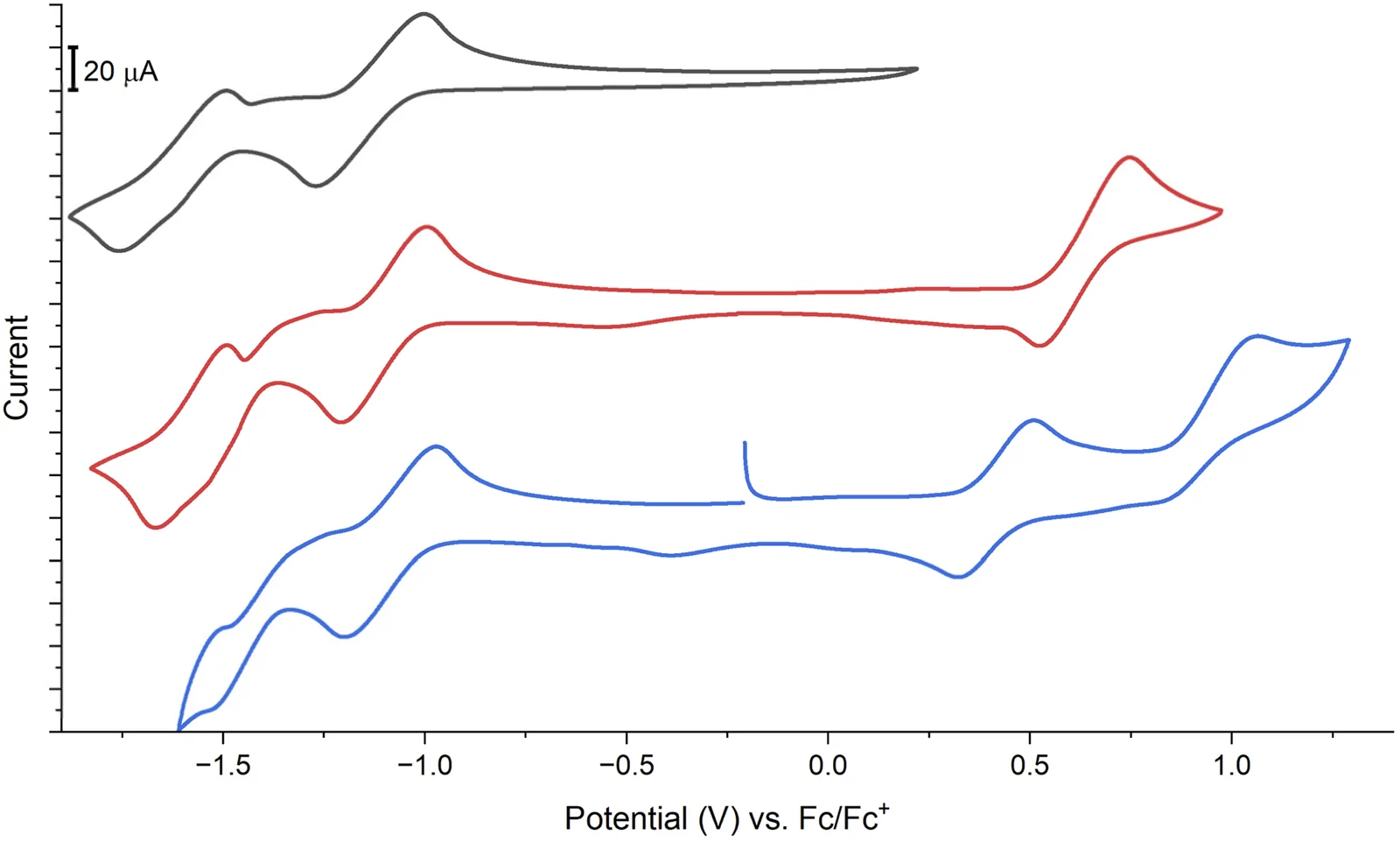
Cyclic voltammograms of NDI-1 (black), NDI-2 (red) and NDI-3 (blue) registered in 0.1 M Bu_4_NClO_4_ in propylene carbonate. Scan rate: 100 mV s^−1^.

**Table 1:** Electrochemical data of compounds NDI-1–NDI-3

	NDI-1	NDI-2	NDI-3
*E*_red._ NDI → NDI˙^−^ [V]	−1.27	−1.20	−1.19
*E*_ox._ NDI˙^−^ → NDI [V]	−1.0	−0.99	−0.97
*E*_1/2_ NDI/NDI˙^−^ [V]	−1.14	−1.1	−1.08
*E*_red._ NDI˙^−^ → NDI^2−^ [V]	−1.75	−1.66	−1.51
*E*_ox._ NDI^2−^ → NDI˙^−^ [V]	−1.49	−1.49	−1.50
*E*_1/2_ NDI˙^−^/NDI^2−^ [V]	−1.62	−1.57	−1.50
*E*_ox._ TPA → TPA˙^+^ [V]	—	+0.74	+0.50
*E*_red._ TPA˙^+^ → TPA [V]	—	+0.52	+0.33
*E*_1/2_ TPA/TPA˙^+^ [V]	—	+0.63	+0.42
*E*_ox._ (Th-TPA)˙^+^ → (Th-TPA)^2+^ [V]	—	—	+1.05
*E*_red._ (Th-TPA)^2+^ → (Th-TPA)˙^+^ [V]	—	—	+0.84
*E*_1/2_ (Th-TPA)˙^+^/(Th-TPA)^2+^ [V]	—	—	+0.95

NDI-2 and NDI-3 were also found to undergo reversible oxidation upon application of a positive potential. For NDI-2, a single electrooxidation event was observed at +0.74 V, which was attributed to the redox activity of the triphenylamine moiety and the formation of the corresponding radical cation. In contrast, NDI-3 displayed two distinct oxidation processes at +0.50 V and +1.05 V, assigned to the oxidation of the triphenylamine and thiophene units, respectively. The shift of the triphenylamine oxidation potential toward less positive values is consistent with observations in related systems, where the introduction of electron-donating substituents facilitates oxidation of the compounds.^32–34^ This behavior reflects the increased ease of charge removal due to the electron-rich nature of the triphenylamine unit and highlights how strategic functionalization can be employed to tune the redox properties of conjugated molecules. In the case of NDI-3, the presence of the additional thiophene unit further modulates the electronic structure, giving rise to two distinct oxidation processes and illustrating the combined effect of multiple electron-donating substituents on the overall electrochemical response.

The frontier molecular orbitals of NDI-1–NDI-3 were analyzed using a combination of cyclic voltammetry and UV-vis spectroscopy. For all compounds, the LUMO energies were determined from the onset of the first reduction wave using the equation *E*_LUMO_ = −(*E*_[onset,red *vs.* Fc^+^/Fc]_ + 4.8) eV. The onset reduction potential slightly increased with increasing electron-donating strength of *N*-substituents (Table 2). Consequently, the calculated LUMO energy levels are −3.77 eV for NDI-1, −3.79 eV for NDI-2 and −3.81 eV for NDI-3. These values indicate only a subtle stabilization of the LUMO level across the series, consistent with the minor variations in the reduction onset potentials. The HOMO energies of NDI-2 and NDI-3 were analogously estimated from the onset of the first oxidation wave, yielding values of −5.34 eV for NDI-2 and −5.14 eV for NDI-3. While NDI-2 and NDI-3 exhibit reversible oxidation, allowing direct determination of their HOMO levels, NDI-1 does not undergo electrochemical oxidation under the applied conditions. Therefore, its HOMO energy was derived from the optical band gap obtained from the onset of the lowest-energy absorption in the UV-vis spectrum, combined with the LUMO energy estimated from the first reduction wave. The optical band gap (Δ*E*_opt._) was calculated according to Δ*E*_opt._ = 1240/*λ*_onset_ [eV], where *λ*_onset_ is the absorption onset wavelength; it was determined to be 3.18 eV, resulting in a HOMO level of −6.96 eV. The calculated band gaps decrease systematically from NDI-1 to NDI-3, reflecting the possibility of modulating the HOMO–LUMO separation through the introduction of electron-donating *N*-substituents, despite the LUMO level remaining nearly unchanged.

**Table 2:** Electrochemical data and calculated HOMO/LUMO energy levels of NDI-1–NDI-3

	NDI-1	NDI-2	NDI-3
*E*_onset red_ [V]	−1.03	−1.01	−0.99
*E*_onset ox_ [V]	—	+0.54	+0.34
HOMO [eV]	−6.96^a^	−5.34	−5.14
LUMO [eV]	−3.77	−3.79	−3.81
Band gap [eV]	3.18^b^	1.55^c^	1.33^c^

a Calculated from the equation: HOMO = LUMO−Δ*E*_opt._.

b Optical band gap.

c Electrochemical band gap.

### Electrochromic properties

Having established the electrochemical behavior of the NDI derivatives, we next examined their electrochromic properties to explore how the observed redox processes translate into optical changes. Spectroelectrochemical measurements were performed to monitor *in situ* the optical response of the materials under varying applied potentials. Measurements were done in 0.1 M Bu_4_NClO_4_/PC as the supporting electrolyte using honeycomb electrode and non-aqueous Ag/Ag^+^ reference electrode. The spectroelectrochemical data of NDI-1–NDI-3 are shown in Fig. 4, 5 and S2. In the neutral state, all compounds exhibited absorption bands at 360 and 380 nm, assigned to π–π* transitions, with a negligible red shift of 2 nm observed for NDI-3. Additionally, NDI-3 displayed an absorption shoulder extending to 440 nm, which may be attributed to the presence of a more conjugated *N*-substituent. Due to their absorption being confined to the UV region, all compounds remained colorless in solution.

**Fig. 4:**
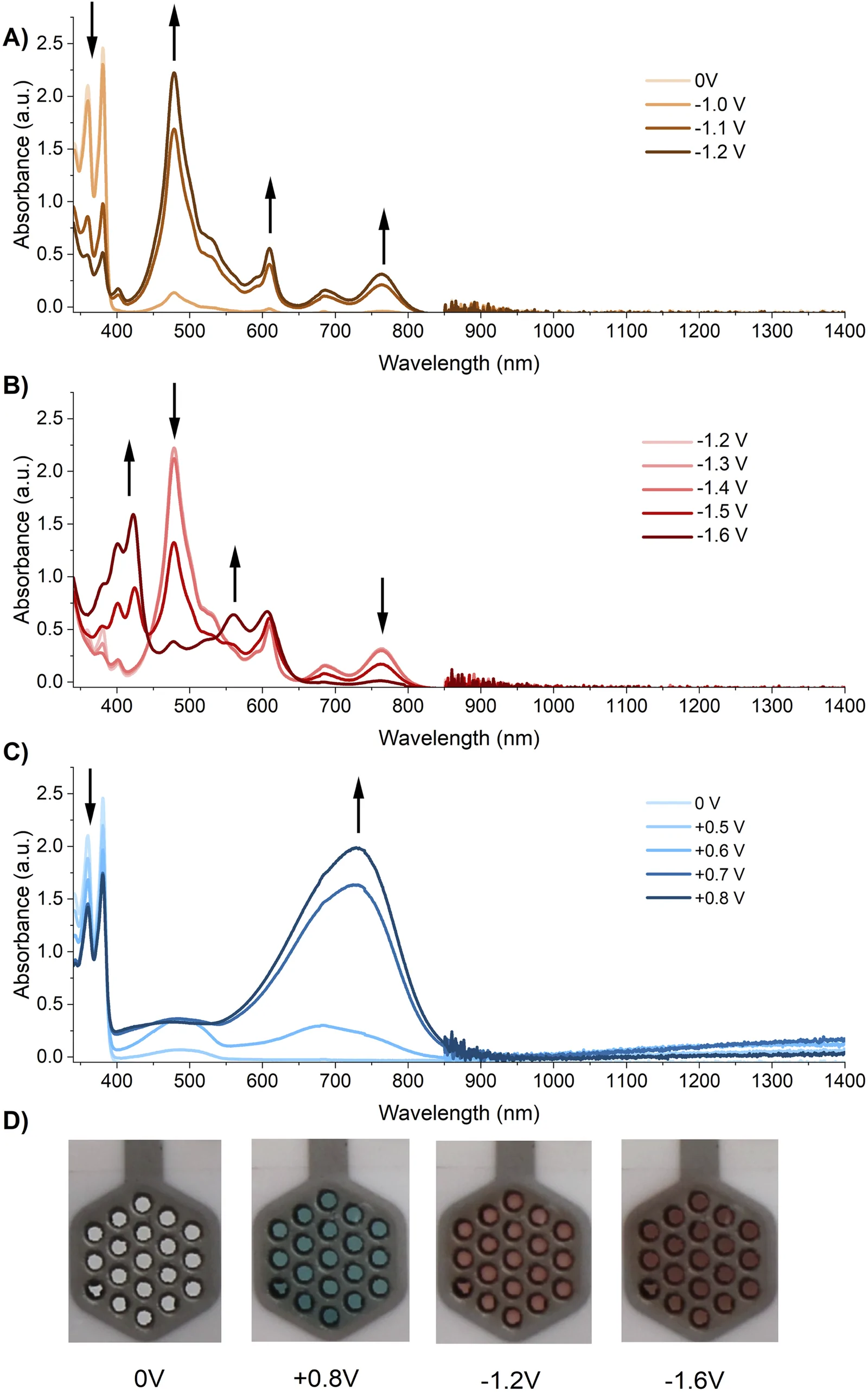
Spectroelectrochemistry of NDI-2 measured in 0.1 M solution of Bu_4_NClO_4_ in anhydrous and deaerated PC as a supporting electrolyte with applied appropriate potential *vs.* Fc/Fc^+^ between (A) 0 V and −1.2 V; (B) −1.2 V and −1.6 V; (C) 0 V and +0.8 V; (D) photographs showing NDI-2 at different redox states.

**Fig. 5:**
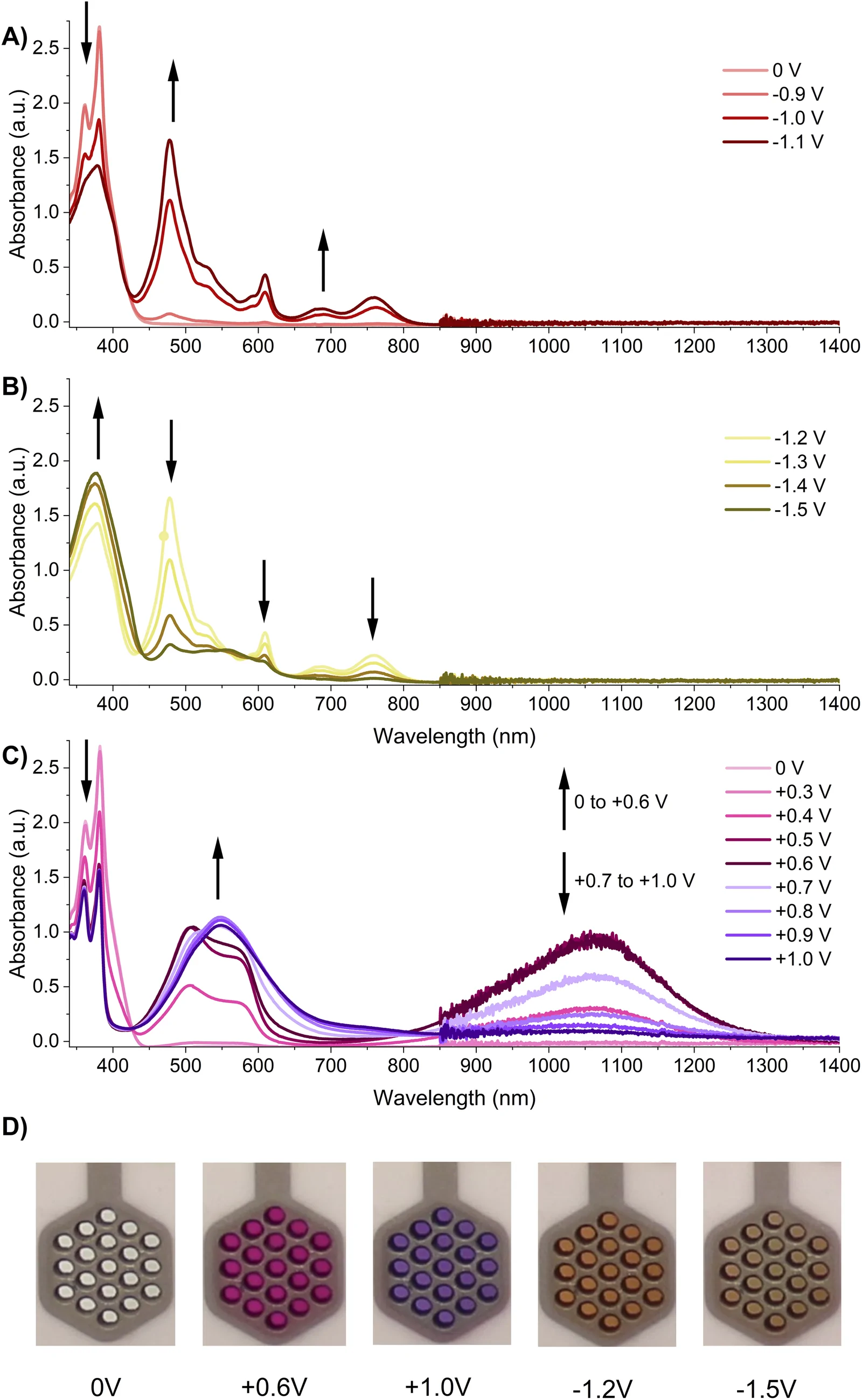
Spectroelectrochemistry of NDI-3 measured in 0.1 M solution of Bu_4_NClO_4_ in anhydrous and deaerated PC as a supporting electrolyte with applied appropriate potential *vs.* Fc/Fc^+^ between (A) 0 V and −1.1 V; (B) −1.2 V and −1.5 V; (C) 0 V and +1.0 V; (D) photographs showing NDI-3 at different redox states.

Upon stepping the potential into the negative range, the compounds exhibited behavior typical of NDI-based materials, forming a radical anion in the first reduction step, followed by the dianion. Formation of the radical anion was indicated by a decrease in the intensity of the UV absorption bands and the emergence of a new dominant transition at 478 nm, accompanied by weaker long-wavelength features at approximately 609, 685, and 760 nm. This pronounced spectral reconfiguration resulted in a color change to orange-red.

Further decreasing the applied potential led to a decrease in the intensity of these bands, and new absorption features appeared in the green-to-red portion of the visible spectrum. Dianions of NDI-1 and NDI-2 exhibited absorption bands at 400, 423, 559, and 607 nm, whereas NDI-3 showed a strong and broad absorption band centered at 376 nm, extending to 450 nm, together with additional bands in the 450–650 nm region. As shown in Fig. S3, the dianions of NDI-1–NDI-3 displayed different absorption in the red and green regions of the spectrum, resulting in different solution colors: gray-green for NDI-1 and brown for both NDI-2 and NDI-3. This observation is consistent with the general trend that green materials exhibit selective absorption with distinct bands in the red and blue regions, whereas brown materials show broad, featureless absorption across most of the visible range, leading to lower color purity. When a slightly oxidizing potential was applied (+0.1 V), the system reverted to its neutral state, as expected for a fully reversible redox process.

NDI-1 did not exhibit an electrooxidation process within the investigated potential window. The oxidation of its phenyl-containing substituent is expected to occur at more positive potentials and is therefore most likely beyond the potential range investigated in this study. Consequently, no additional oxidized state of NDI-1 could be generated, and no anodically induced color change was observed. The introduction of electron-donating triphenylamine-based substituents in NDI-2 and NDI-3, in contrast, resulted in accessible oxidation processes and pronounced changes in their optical response, highlighting the important role of the *N*-substituent in controlling the redox and electrochromic behavior of the NDI derivatives. For NDI-2, applying the oxidizing potential led to the formation of the triphenylamine radical cation,^35,36^ which was detected as the appearance of a strong and broad absorption band with a maximum at 730 nm, corresponding to a color change from colorless to blue. For NDI-3, increasing the potential within the oxidizing range up to +0.6 V resulted in the emergence of two distinct absorption features in the visible region, with a main band at 509 nm and a shoulder at 564 nm, accompanied by a broad and intense NIR absorption centered at 1064 nm. The simultaneous appearance of these transitions is consistent with the generation of a delocalized radical cation species,^37–40^ which manifests visually as a clear color change from colorless to purple. Further oxidation led to the formation of the dication, a transformation reflected in the disappearance of the NIR band and a bathochromic shift of the visible absorption maximum to 550 nm. This spectral evolution correlates with a second distinct color transition – from purple to a deep blue-violet – indicating the progression from the monocation radical to the fully oxidized dicationic state.

To facilitate a direct comparison of the electrochromic behavior of NDI-1–NDI-3, the applied potentials, corresponding redox states, characteristic absorption features, and observed solution colors are summarized in Table 3. The comparison clearly demonstrates the progressive increase in the number of accessible optical states upon increasing the electron-donating character and conjugation of the aromatic *N*-substituent.

**Table 3:** Summary of the spectroelectrochemical response and optical states of NDI-1–NDI-3 in solution at selected applied potentials

Compound	Applied potential *vs.* Fc/Fc^+^ (V)	Redox state	Characteristic absorption bands (nm)	Observed color
NDI-1	0.0	Neutral	360, 380	Colorless
	−1.0 to −1.2	Radical anion	∼478; ∼609, 685, 760	Orange-red
	−1.3 to −1.6	Dianion	400, 423, 559, 607	Gray-green
NDI-2	0.0	Neutral	360, 380	Colorless
	−1.0 to −1.2	Radical anion	∼478; ∼609, 685, 760	Orange-red
	−1.3 to −1.6	Dianion	400, 423, 559, 607	Brown
	+0.5 to +0.8	Radical cation	∼730	Blue
NDI-3	0.0	Neutral	360, 380; shoulder to ∼440	Colorless
	−0.9 to −1.2	Radical anion	∼478; ∼609, 685, 760	Orange-red
	−1.3 to −1.5	Dianion	∼376–450; additional bands 450–650	Brown
	+0.3 to +0.6	Radical cation	509, 564; 1064	Purple
	+0.7 to +1.0	Dication	∼550	Blue-violet

The NDI derivatives exhibit distinct color changes upon sequential electrochemical reduction and oxidation. NDI-1 displays three colors, corresponding to its neutral, radical anion, and dianion states. NDI-2 undergoes four color transitions, reflecting the formation of radical anions, dianions, and the electrochemically generated triphenylamine radical cation. NDI-3 shows the richest color palette, exhibiting five distinct colors across its redox states, due to the combined effects of its radical anion, dianion, and oxidized species, along with strong and broad absorption features in the visible and near-infrared regions. These observations highlight the tunable optical properties of NDI derivatives and their potential for multistate electrochromic applications.

### Multielectrochromic device

Since NDI-3 exhibited the highest number of accessible redox states, each accompanied by a distinct optical response, it was selected as the active material for the fabrication of the multielectrochromic device. The device was assembled using glass substrates (2.5 × 2.0 cm) coated with indium-tin oxide (ITO, *R*_s_ = 8–12 Ω) serving as transparent electrodes. An NDI-3 film was spray-coated onto the first ITO substrate from 1 mL of a 2 mg mL^−1^ solution and then assembled with a second ITO substrate coated with an ion-conducting gel electrolyte prepared as described previously.^41,42^ A propylene carbonate-based gel was selected to ensure consistency with the color changes observed during the solution-phase spectroelectrochemical measurements. Electrical contact to the potentiostat was established using copper tape.

The electrochromic properties of the assembled device were systematically investigated under varying applied potentials to gain a detailed understanding of its optical response and switching behavior. This approach allowed for a comprehensive assessment of the material's ability to undergo reversible color changes over multiple redox cycles, the speed at which these changes occur, and the overall coloration efficiency, providing valuable insights into its potential performance in practical electrochromic applications. It was observed that the device containing NDI-3 as the active material undergoes a two-step reduction as well as a two-step oxidation, which is consistent with the spectroelectrochemical behavior of this compound recorded in solution. As shown in Fig. 6, the optical properties of the device were evaluated in transmittance mode over the range of 400–1500 nm.

**Fig. 6:**
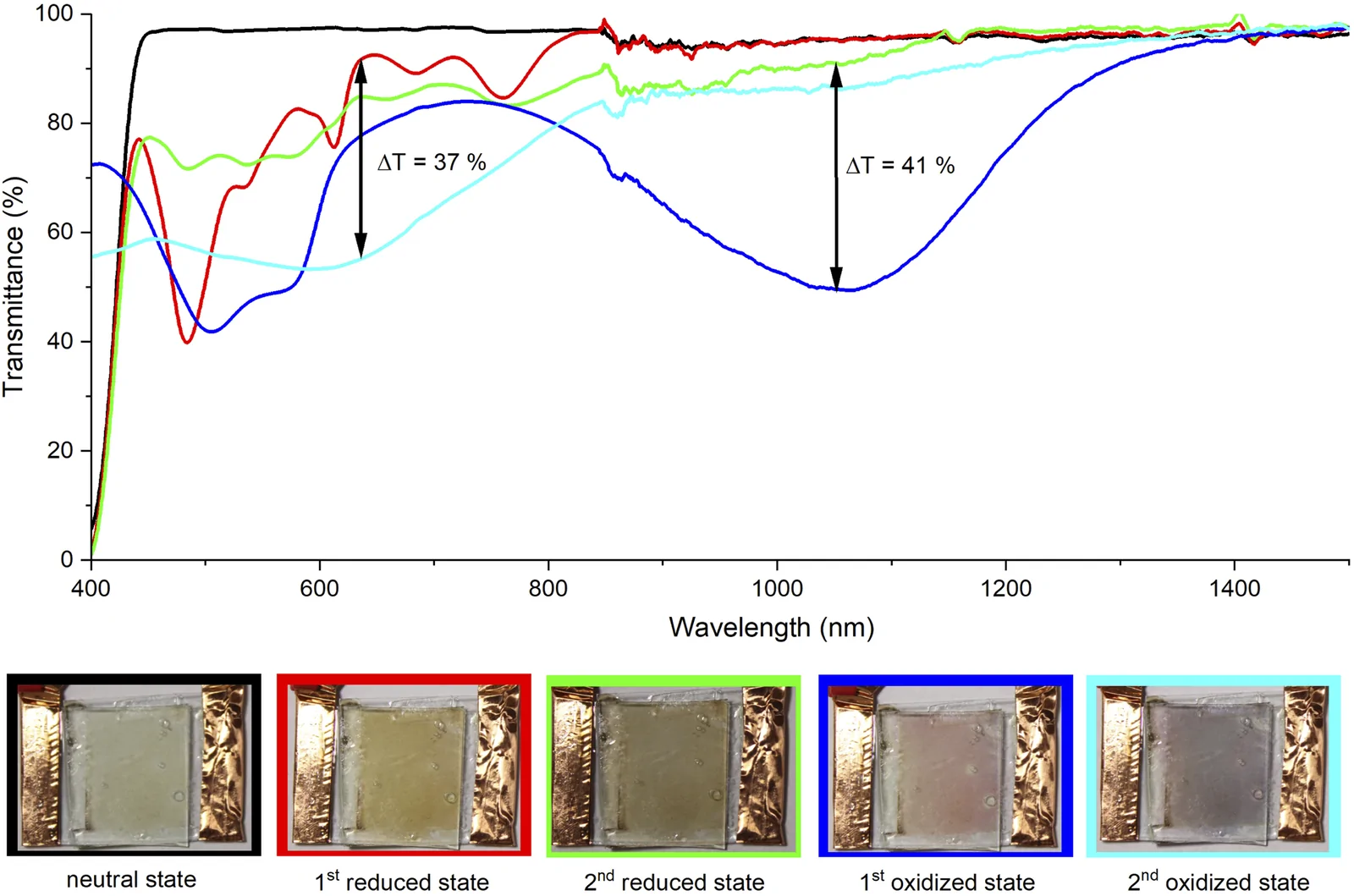
The transmission spectra of the device fabricated with NDI-3 were recorded after applying potentials of 0 V (black), −1.4 V (
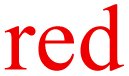
), −1.6 V (
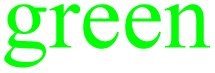
), +0.9 V (
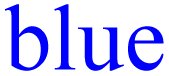
), and +1.2 V (
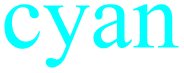
), along with photographs showing the device in its different redox states.

The device containing NDI-3 in its neutral state exhibited high transmittance across almost the entire visible and near-infrared (NIR) regions. Upon applying a reduction potential of −1.4 V, a color change from yellowish to orange was observed. Further reduction at −1.6 V resulted in the device turning brown. Conversely, applying a positive potential of +0.9 V induced a color change to red, accompanied by a decrease in transmittance in the 420–640 nm range as well as in the NIR region (800–1300 nm). Further increase in applied potential led to a color change to blue-violet. Although the color changes observed for the solid-state device are less pronounced visually than those observed in the solution-phase spectroelectrochemical measurements, the device exhibits clear and reversible optical modulation upon application of different potentials. The relatively subtle differences in the photographs may be related to the amount and optical density of the deposited NDI-3 layer, as well as to the device configuration and photographic conditions. Further optimization of the active-layer loading and device architecture could therefore enhance the visual color contrast between the different redox states. Nevertheless, the electrochromic response of the device is clearly demonstrated by the corresponding transmittance changes.

The electrochromic stability of the device was systematically evaluated by repetitive potential switching between the brown and red states, corresponding to the dianion-to-radical cation transition, and between the orange and blue-violet states, associated with the radical anion-to-dication redox process. The measurements were performed under dynamic conditions, with consecutive switching cycles and a residence time of 10 s at each applied potential, in order to assess both the optical contrast retention and long-term operational durability of the system.

For the brown ↔ red transition, the potential was alternated between −1.6 V and +0.9 V to induce the reversible interconversion between the dianion and radical cation forms. The initial transmittance difference (Δ*T*) between the brown and red states, measured in the near-infrared (NIR) region at 1050 nm, was 41%, indicating a high optical contrast in this spectral range. Upon prolonged cycling, a gradual decrease in Δ*T* was observed, reaching approximately 13% after more than 400 oxidation–reduction cycles. As shown in Fig. 7A and B, the transmittance of the dianion form (brown state) progressively changes over time, suggesting partial instability or degradation of the dianion species under repeated redox conditions. This behavior may be associated with structural or interfacial changes occurring during extended electrochemical operation. Nevertheless, despite the noticeable decrease in optical contrast, the device maintains reversible switching and a distinguishable color modulation, demonstrating satisfactory electrochromic stability for the brown ↔ red transition.

**Fig. 7:**
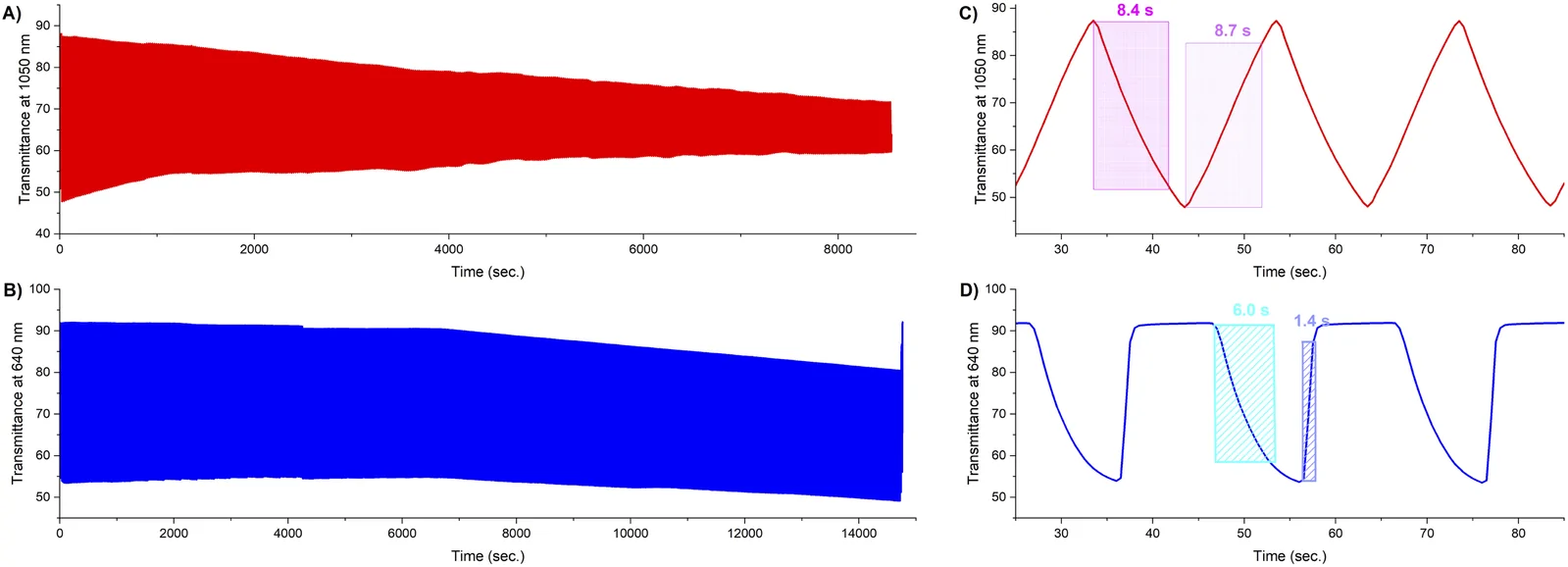
The electrochromic stability of the device when switched between (A) −1.6 V and +0.9 V; (B) −1.4 V and +1.2 V; (C) switching times between brown and red states; (D) switching times between orange and blue-violet states.

In the case of the orange ↔ blue-violet transition, the potential was switched between −1.4 V and +1.2 V, and the transmittance difference was monitored at 640 nm, corresponding to the characteristic absorption changes of the radical anion and dication forms. The initial Δ*T* between these states was 37%, reflecting a pronounced and visually distinct color change. Notably, after more than 700 redox cycles, the transmittance difference decreased by only 6 percentage points, reaching 31%. This minor reduction in optical contrast over an extended number of cycles indicates significantly improved cycling stability for this redox couple compared to the brown ↔ red process.

Overall, the results demonstrate that the device exhibits good to very good electrochemical and electrochromic performance, with particularly high stability for the radical anion ↔ dication transition. The retained optical contrast after several hundred switching cycles confirms the robustness of the redox-active system and its potential applicability in durable electrochromic devices.

Switching time is another key parameter used to characterize the performance of electrochromic devices. It is defined as the time required to achieve 90% of the total optical change associated with a given color transition. This parameter reflects the kinetics of the redox processes and ion transport within the electrochromic layer and, consequently, determines the practical responsiveness of the device. As shown in Fig. 7C and D, the coloration time for the brown-to-red transition was 8.4 s, while the reverse red-to-brown bleaching process required 8.7 s. In contrast, for the orange ↔ blue-violet transition, faster switching was observed. The coloration time for the orange-to-blue-violet process was 6.0 s, whereas the reverse blue-to-orange transition occurred significantly more rapidly, within 1.4 s. Overall, the relatively short switching times, particularly for the orange ↔ blue-violet transition, confirm the good dynamic response of the device and further support its potential applicability in practical electrochromic systems.

Coloration efficiency (CE) was determined to further evaluate the electrochromic performance of the NDI-3-based device. The coloration efficiency was calculated according to CE = ΔOD/*Q*_d_, where ΔOD is the change in optical density, calculated as ΔOD = log(*T*_b_/*T*_c_), and *Q*_d_ is the charge density required to induce the corresponding optical change. For the brown ↔ red transition, the optical modulation was evaluated at 1050 nm, whereas the orange ↔ blue-violet transition was analyzed at 640 nm. The resulting CE values for both redox transitions are summarized in Table 4. The calculated coloration efficiencies were 85 and 120 cm^2^ C^−1^ for the brown ↔ red and orange ↔ blue-violet transitions, respectively. The higher CE observed for the orange ↔ blue-violet transition indicates that a larger optical change is achieved per unit of injected charge, demonstrating more efficient utilization of the electrochemically generated charge for optical modulation. These results, together with the high transmittance modulation and short switching times, further demonstrate the potential of the NDI-3-based device for multistate electrochromic applications.

**Table 4:** Electrochromic performance parameters of the NDI-3-based device for the two investigated color transitions

Transition	Potential range (V)	*λ* (nm)	Δ*T* (%)	ΔOD	*Q*_d_ (mC cm^−2^)	CE (cm^2^ C^−1^)	Switching time (s)^a^
Brown ↔ red	−1.6/+0.9	1050	41	0.27	3.18	85	8.4/8.7
Orange ↔ blue-violet	−1.4/+1.2	640	37	0.22	1.87	120	6.0/1.4

a Switching times are given as coloration/bleaching times.

To place the electrochromic properties of the NDI derivatives investigated in this work in the context of previously reported materials, a comparison with representative NDI-based electrochromic systems is presented in Table 5.

**Table 5:** Comparative electrochromic performance of representative NDI-based materials

Material	Electrochromic states	Δ*T* (%)	CE (cm^2^ C^−1^)	Switching time, coloration/bleaching (s)	Cycling stability	Ref.
Thiophene-NDI donor–acceptor polymer^a^^,^^b^	3 colors	23% (770 nm)	82	1.4/0.7	77% after 1000 cycles	43
Ni-NDI MOF^a^^,^^c^	3 colors	73% (720 nm)	260	2.1/1.9	>500 cycles	44
NDI-cored pyridinium salt (Me2DPNDI) (2I)^e^	2 colors	57.2% (446 nm)	199	2.32/3.04	<4% change in optical contrast after 7200 s	45
TPA–iso-NDI (p-TPA2ISO) polymer^a^^,^^d^	3 colors	51.4% (740 nm)	203	1.28/0.73	Up to 500 cycles; ∼10% activity loss after 3000 s	46
NDI-based styrene polymer^f^	3 colors	26% (625 nm)	238	18/6	∼20% Δ*T* retained after 500 cycles	24
NDI-3 (this work)^f^	5 colors	41% (1050 nm)	85	8.4/8.7	13% after more than 400 redox cycles	This work
		31% (640 nm)	120	6.0/1.4	31% after 700 redox cycles	

a Deposited on ITO; measured in.

b 0.1 M TBAPF_6_/CH_3_CN.

c 0.1 M NaClO_4_/PC.

d 0.1 M TBAP in CH_3_CN.

e Liquid-state sandwich-type electrochromic device.

f Solid-state electrochromic device.

As shown in Table 5, NDI-based electrochromic materials can exhibit high optical contrast, high coloration efficiency, fast switching, or multistate electrochromism. However, these properties are often achieved through polymerization, incorporation into extended frameworks, or structural modification involving additional electroactive units. In the present work, we demonstrate that systematic modification of the aromatic *N*-substituent within a single unsymmetrical NDI molecular scaffold provides a straightforward means of progressively increasing the number of accessible electrochromic states, from three for NDI-1 to four for NDI-2 and five for NDI-3.

## Conclusions

Three unsymmetrical naphthalene diimide derivatives with systematically varied aromatic *N*-substituents were successfully synthesized and investigated as multistate electrochromic materials. The results demonstrate that molecular engineering of the *N*-substituent provides an effective approach to controlling the redox behavior, frontier orbital energies, and optical response of NDI-based systems. Increasing the electron-donating strength of the aromatic substituent enabled additional reversible oxidation processes while preserving the characteristic two-step reduction of the NDI core, resulting in progressively richer electrochromic behavior with up to five accessible optical states.

Among the investigated compounds, NDI-3 exhibited the broadest electrochemical activity and the most pronounced modulation of both visible and near-infrared absorption. Its integration into a solid-state electrochromic device confirmed reversible multicolor switching, transition-dependent cycling stability, with particularly high stability observed for the radical anion/dication transition, and distinct optical modulation, while further optimization of the device architecture could improve its visual color contrast and switching performance.

These findings establish unsymmetrical NDI derivatives as a versatile molecular platform for designing multistate electrochromic materials. The demonstrated ability to tailor optical response through rational molecular design offers new opportunities for advanced electrochromic devices requiring controllable visible and near-infrared light modulation, including energy-efficient smart window technologies.

## Conflicts of interest

There is no conflict of interests.

## Supplementary Material

RA-OLF-D6RA08357A-s001

## Data Availability

The data supporting this article have been included as part of the supplementary information (SI). Supplementary information: experimental section, spectroelectrochemistry of NDI-1, UV-vis spectra of dianions, NMR spectra, ESI-MS spectra. See DOI: https://doi.org/10.1039/d6ra08357a.
