# Supplementary material for: Unsymmetrical naphthalene diimides as electrochromic materials: tailoring structure for controlled optical and redox properties
Source: RSC Adv. 2026 Oct 6;16(46):60358–69. doi: 10.1039/d6ra08357a (PMC13639873; doi:10.1039/d6ra08357a)
Supplement: Supplementary file 1 [file RA-016-D6RA08357A-s001.pdf]

Supplementary Information (SI) for RSC Advances.
This journal is © The Royal Society of Chemistry

Supplementary information

Unsymmetrical Naphthalene Diimides as Electrochromic Materials: Tailoring Structure for Controlled Optical and Redox Properties

Marcin Nowacki, Sergiusz Napierała, Monika Wałęsa-Chorab*

Faculty of Chemistry, Adam Mickiewicz University in Poznań, Uniwersytetu Poznańskiego 8, 61-614 Poznań, Poland

* *Correspondence:* mchorab@amu.edu.pl

Table of content

Figure S 1. Synthetic scheme of **A** and **NDI-1** – **NDI-3**. 7

Figure S 2. Spectroelectrochemistry of **NDI-1** measured in 0.1 M solution of $Bu4NClO_4$ in anhydrous and deaerated PC as a supporting electrolyte with applied appropriate potential vs Fc/Fc^+ between A) 0 V and -1.2 V; B) -1.2 V and -1.5 V; C) Photographs showing **NDI-1** at different redox states. 8

Figure S 3. Normalized absorption spectra of dianions of **NDI-1** (■), **NDI-2** (●) and **NDI-3** (▲) showing the absorption in red-green region. 9

Figure S 4. ^{1}H NMR spectra of **A** in DMSO-d_6. 9

Figure S 5. ^{1}H NMR spectra of **C** in $CDCl_3$. 10

Figure S 6. ^{13}C NMR spectra of **C** in $CDCl_3$. 10

Figure S 7. ^{1}H NMR spectra of **NDI-1** in $CDCl_3$. 11

Figure S 8. ^{13}C NMR spectra of **NDI-1** in $CDCl_3$. 11

Figure S 9. ^{1}H NMR spectra of **NDI-2** in $CDCl_3$. 12

Figure S 10. ^{13}C NMR spectra of **NDI-2** in $CDCl_3$. 12

Figure S 11. ^{1}H NMR spectra of **NDI-3** in $CDCl_3$. 13

Figure S 12. ^{13}C NMR spectra of **NDI-3** in $CDCl_3$. 13

Figure S 13. ESI-MS spectra of compound **NDI-1**. 14

Figure S 14. ESI-MS spectra of compound **C**. 14

Figure S 15. ESI-MS spectra of compound **NDI-2**. 15

Figure S 16. ESI-MS spectra of compound **NDI-3**. 16

Experimental section:

Materials and methods:

All chemicals and solvents were purchased from commercial sources (Aldrich and Fluorochem) and were used as received, without additional purification. NMR spectra were acquired on a Bruker Avance NEO 600 MHz spectrometer. Chemical shifts are reported in parts per million (ppm) and were referenced to residual proton signals of the deuterated solvents ($CDCl_3$: 7.26 ppm; DMSO-d_6: 2.50 ppm). High-resolution mass spectrometry measurements were performed in positive ion mode using a QTOF Impact HD instrument (Bruker Daltonics, Bremen, Germany). UV-Vis-NIR absorption spectra were recorded on a Jasco V-770 spectrophotometer. Electrochemical studies were conducted with a multichannel BioLogic VSP potentiostat using an anhydrous 0.1 M Bu_4NClO_4 in propylene carbonate as the supporting electrolyte. A platinum disk served as the working electrode, a platinum wire as the counter electrode, and a non-aqueous Ag/Ag^+ electrode as the reference electrode. Cyclic voltammograms were calibrated against ferrocene/ferrocenium redox couple using ferrocene as an external standard. Spectroelectrochemical experiments were performed using the same potentiostat coupled to a Jasco V-770 UV-Vis-NIR spectrometer. These measurements employed a commercially available honeycomb working electrode, a platinum counter electrode, and a non-aqueous Ag/Ag^+ reference electrode. The Ag/Ag^+ reference electrode was prepared by immersing a cleaned silver wire into a glass capillary with a porous frit filled with a solution containing 0.01 M $AgNO_3$ and 0.1 M TBAP in acetonitrile.

Synthesis of compounds:

*Synthesis of **A**:* 1,4,5,8-Naphthalenetetracarboxylic dianhydride (NDA, 0.8 g, 2.98 mmol) was dissolved in 18 mL of dry DMF in a microwave reaction tube. *N*-Hexylamine (0.302 g, 2.98 mmol) was added dropwise to the solution under stirring. The reaction mixture was then

sonicated for 1 hour. Subsequently, the mixture was heated under microwave irradiation at 60 °C for 40 minutes, followed by 140 °C for 15 minutes. After completion, the reaction mixture was poured into 100 mL of 1 M hydrochloric acid. The resulting solid was collected by filtration under reduced pressure and washed thoroughly with water, affording a yellowish solid (0.95 g, 91%) ^{1}H NMR (400 MHz, DMSO-d_6) δ 13.58 (s, 2H), 8.70 – 8.62 (m, 2H), 8.54 (d, *J* = 7.5 Hz, 2H), 8.18 (d, *J* = 7.5 Hz, 2H), 4.08 – 3.97 (m, 3H), 1.70 – 1.56 (m, 3H), 1.41 – 1.20 (m, 9H), 0.86 (td, *J* = 5.9, 4.9, 3.3 Hz, $4\frac{1}{2}$H) ppm.

Compounds **B**[1] and **D**[2] were synthesized following previously described procedures.

*Synthesis of 4-(5-(4-aminophenyl)thiophen-2-yl)-N,N-diphenylaniline (**C**):* In a 100 mL round-bottom flask, compound **D** (420 mg, 1.03 mmol), 4-aminophenylboronic acid pinacol ester (271 mg, 1.24 mmol), and K_2CO_3 (427 mg, 3.09 mmol) were dissolved in a mixture of toluene (5 mL) and water (10 mL) under an argon atmosphere. The solution was degassed for 20 minutes, after which tetrakis(triphenylphosphine)palladium(0) (59.5 mg, 0.05 mmol) was added. The reaction mixture was then heated at 95 °C for 20 hours. After cooling to room temperature, the mixture was extracted with water and dichloromethane. The combined organic layers were dried over anhydrous magnesium sulfate and concentrated under reduced pressure. The brown solid was obtained by isolation through a chromatographic column with an eluent of 2:1 dichloromethane hexane (200 mg, 46.2%). ^{1}H NMR (600 MHz, $CDCl_3$): δ 7.47 (d, *J* = 8.6 Hz, 2H), 7.43 (d, *J* = 8.5 Hz, 2H), 7.30 – 7.23 (m, 4H), 7.15 (d, *J* = 3.7 Hz, 1H), 7.15 – 7.09 (m, 5H), 7.06 (d, *J* = 8.8 Hz, 2H), 7.03 (tt, *J* = 7.4, 1.1 Hz, 2H), 6.72 (d, *J* = 8.5 Hz, 2H) ppm. ^{13}C NMR (150 MHz, $CDCl_3$): δ 115.6, 122.3, 123.1, 123.2, 124.0, 124.6, 125.5, 126.4, 126.9, 128.9, 129.4, 141.8, 143.6, 145.8, 147.1, 147.7 ppm. HRMS: 419.1584 m/z $[M+H]^+$

*Synthesis of **NDI-1***: In a microwave tube, compound **A** (300 mg, 0.85 mmol) was dissolved in anhydrous DMF (6 mL). Aniline (89 mg, 0.96 mmol) was then added dropwise, followed by

the addition of triethylamine (0.96 mmol). The reaction was carried out in a microwave reactor at 180 °C for 1.5 hours. After completion, the reaction mixture was poured into 100 mL of 1 M hydrochloric acid. The resulting precipitate was collected by centrifugation and washed with water. A light pink solid was obtained (100 mg, 27.5%). ^{1}H NMR (600 MHz, $CDCl_3$): δ 8.80 (d, J = 1.6 Hz, 4H), 7.61 – 7.55 (m, 2H), 7.53 (dd, J = 8.3, 6.6 Hz, 1H), 7.35 – 7.31 (m, 2H), 4.21 (td, J = 7.5, 1.5 Hz, 2H), 1.76 (td, J = 8.6, 4.2 Hz, 2H), 1.44 (q, J = 7.8, 7.2 Hz, 2H), 1.35 (td, J = 8.9, 8.3, 4.7 Hz, 4H), 0.90 (t, J = 6.2 Hz, 3H) ppm. ^{13}C NMR (150 MHz, $CDCl_3$): δ 163.2, 162.9, 134.7, 131.5, 131.2, 129.7, 129.3, 128.6, 127.1, 126.8, 41.2, 31.6, 28.2, 26.9, 22.7, 14.2 ppm. HRMS: 449.1483 m/z $[M+Na]^+$

*Synthesis of **NDI-2***: In a microwave tube, compound **A** (122.85 mg, 0.35 mmol) was dissolved in DMF (3 mL), followed by the addition of compound **B** (100 mg, 0.38 mmol) and triethylamine (0.38 mmol). The reaction mixture was heated in a microwave reactor at 160 °C for 1.5 hours. After completion, the mixture was poured into 1M hydrochloric acid solution, then filtered and washed several times with water. The organic layer was dried over anhydrous $MgSO_4$ and concentrated under reduced pressure, yielding a purple solid (132.4 mg, 62%). ^{1}H NMR (600 MHz, $CDCl_3$): δ 8.80 (d, J = 0.9 Hz, 4H), 7.33 – 7.27 (m, 4H), 7.23 – 7.17 (m, 6H), 7.17 – 7.12 (m, 2H), 7.11 – 7.05 (m, 2H), 4.24 – 4.18 (m, 2H), 1.80 – 1.71 (m, 2H), 1.49 – 1.41 (m, 2H), 1.40 – 1.32 (m, 4H), 0.90 (t, J = 7.0 Hz, 3H) ppm. ^{13}C NMR (150 MHz, $CDCl_3$): δ 163.4, 162.9, 147.5, 131.5, 131.2, 129.6, 129.1, 127.1, 126.9, 125.4, 123.8, 122.9, 41.2, 31.6, 28.2, 26.9, 22.7, 14.2 ppm. ESI-MS: 594.24. HRMS: 594.2390 m/z $[M+H]^+$

***Synthesis of NDI-3*:** In a microwave tube, product **C** (180 mg, 0.43 mmol) and product **A** (137 mg, 0.39 mmol) were dissolved in anhydrous DMF (3 mL). The reaction was carried out in a microwave reactor at 180 °C for 1.5 hours. After this time, the mixture was evaporated under reduced pressure, and the crude product was purified by column chromatography using dichloromethane as eluent. A green precipitate was obtained (169 mg, 57.6%). ^{1}H NMR (600

MHz, $CDCl_3$): δ 8.84 – 8.78 (m, 4H), 7.80 (d, *J* = 8.5 Hz, 2H), 7.51 (d, *J* = 8.7 Hz, 2H), 7.34 (dd, *J* = 6.2, 2.4 Hz, 3H), 7.28 (dd, *J* = 8.6, 7.3 Hz, 4H), 7.23 (d, *J* = 3.8 Hz, 1H), 7.16 – 7.11 (m, 4H), 7.09 (d, *J* = 8.7 Hz, 2H), 7.05 (tt, *J* = 7.4, 1.2 Hz, 2H), 4.25 – 4.19 (m, 2H), 1.81 – 1.74 (m, 2H), 1.49 – 1.41 (m, 2H), 1.40 – 1.33 (m, 4H), 0.91 (t, *J* = 7.0 Hz, 3H) ppm. ^{13}C NMR (150 MHz, $CDCl_3$): δ 163.1, 162.9, 147.6, 131.6, 131.2, 131.1, 129.5, 129.1, 128.2, 127.1, 126.8, 126.7, 126.6, 125.1, 124.7, 123.4, 123.3, 41.1, 31.6, 28.2, 27.0, 22.8, 14.2 ppm. HRMS: 774.2419 m/z $[M+Na]^+$

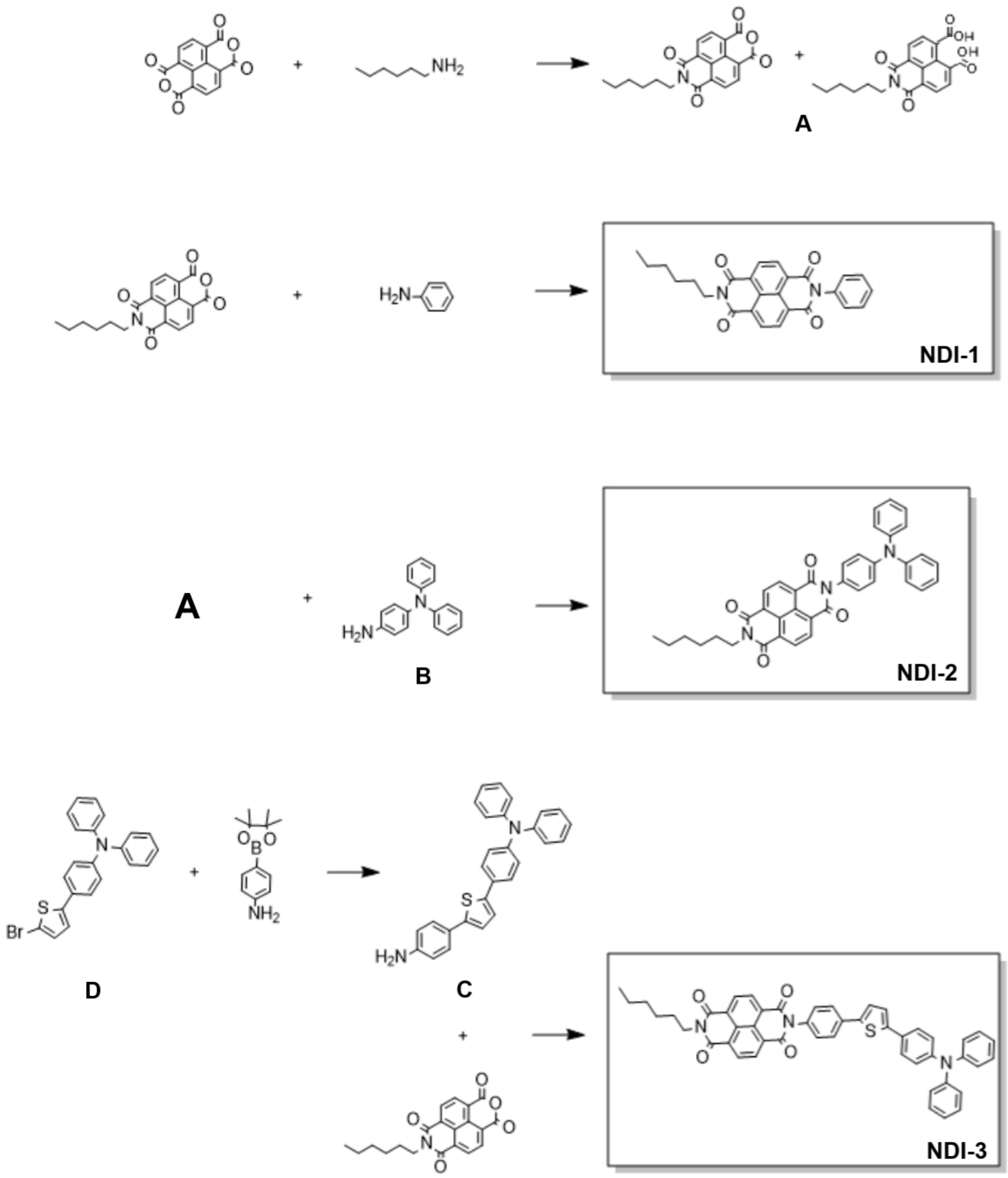

Figure S 1. Synthetic scheme of **A** and **NDI-1** – **NDI-3**.

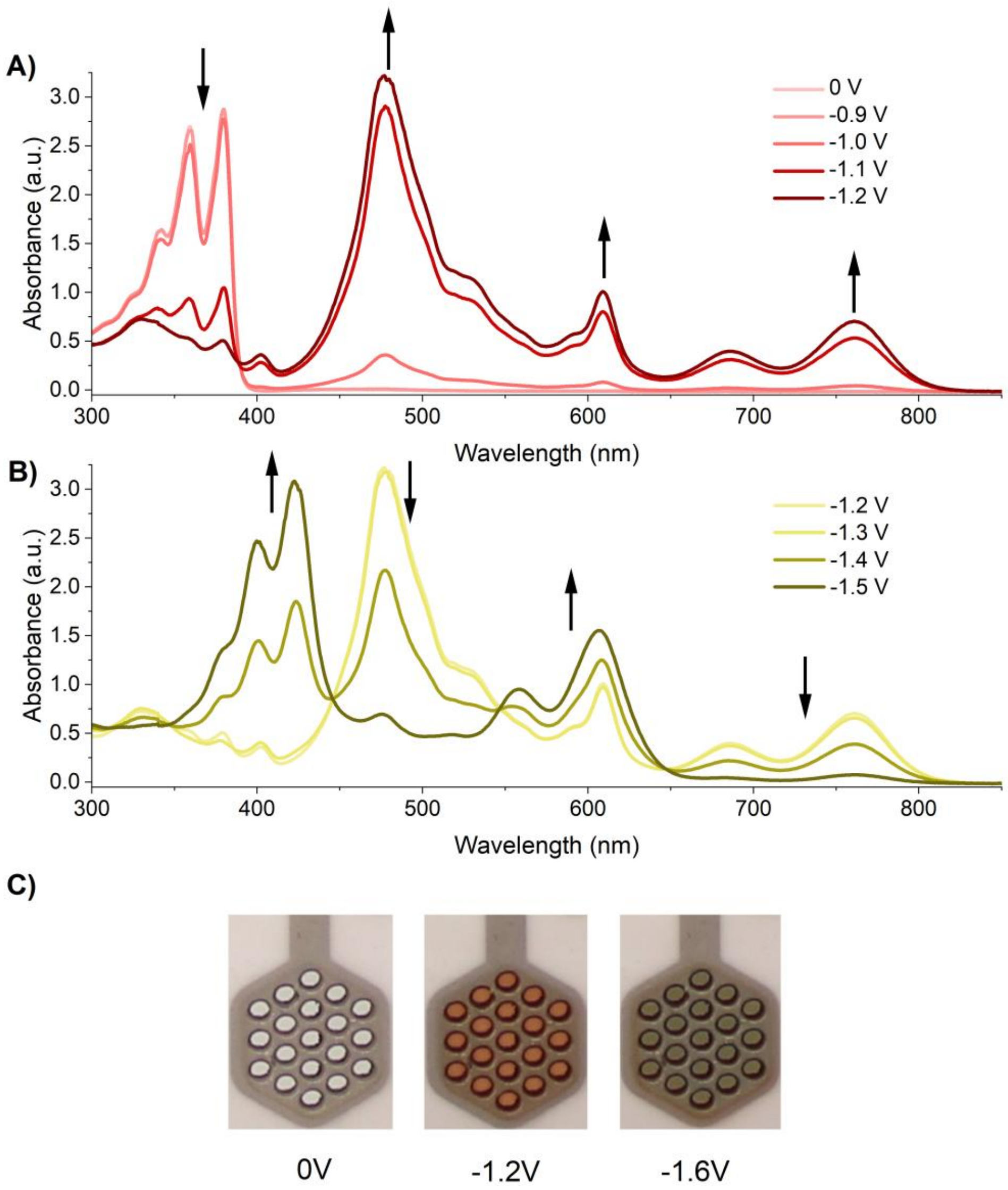

Figure S 2. Spectroelectrochemistry of **NDI-1** measured in 0.1 M solution of Bu_4NClO_4 in anhydrous and deaerated PC as a supporting electrolyte with applied appropriate potential vs Fc/Fc^+ between A) 0 V and -1.2 V; B) -1.2 V and -1.5 V; C) Photographs showing **NDI-1** at different redox states.

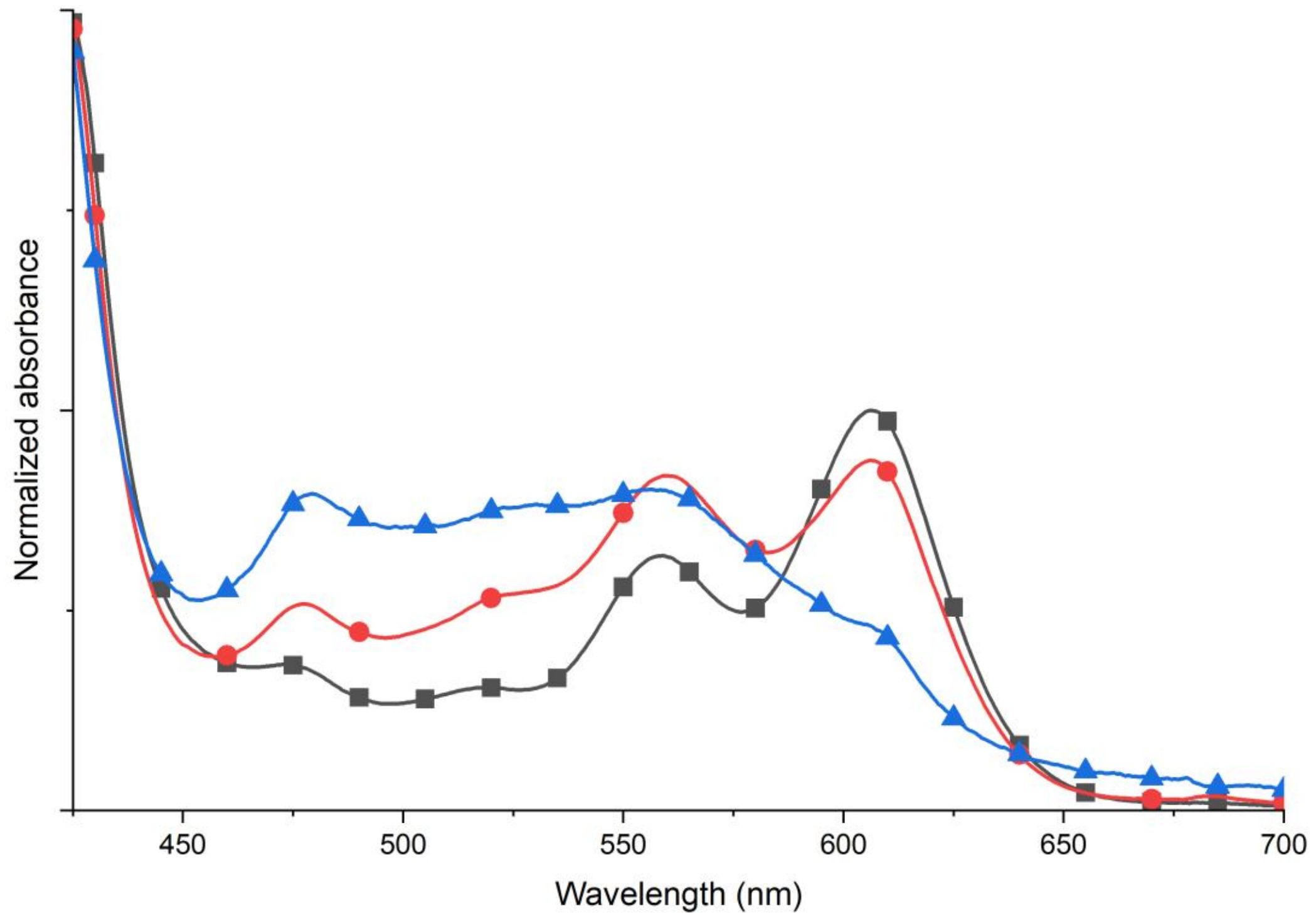

Figure S 3. Normalized absorption spectra of dianions of **NDI-1** (■), **NDI-2** (●) and **NDI-3** (▲) showing the absorption in red-green region.

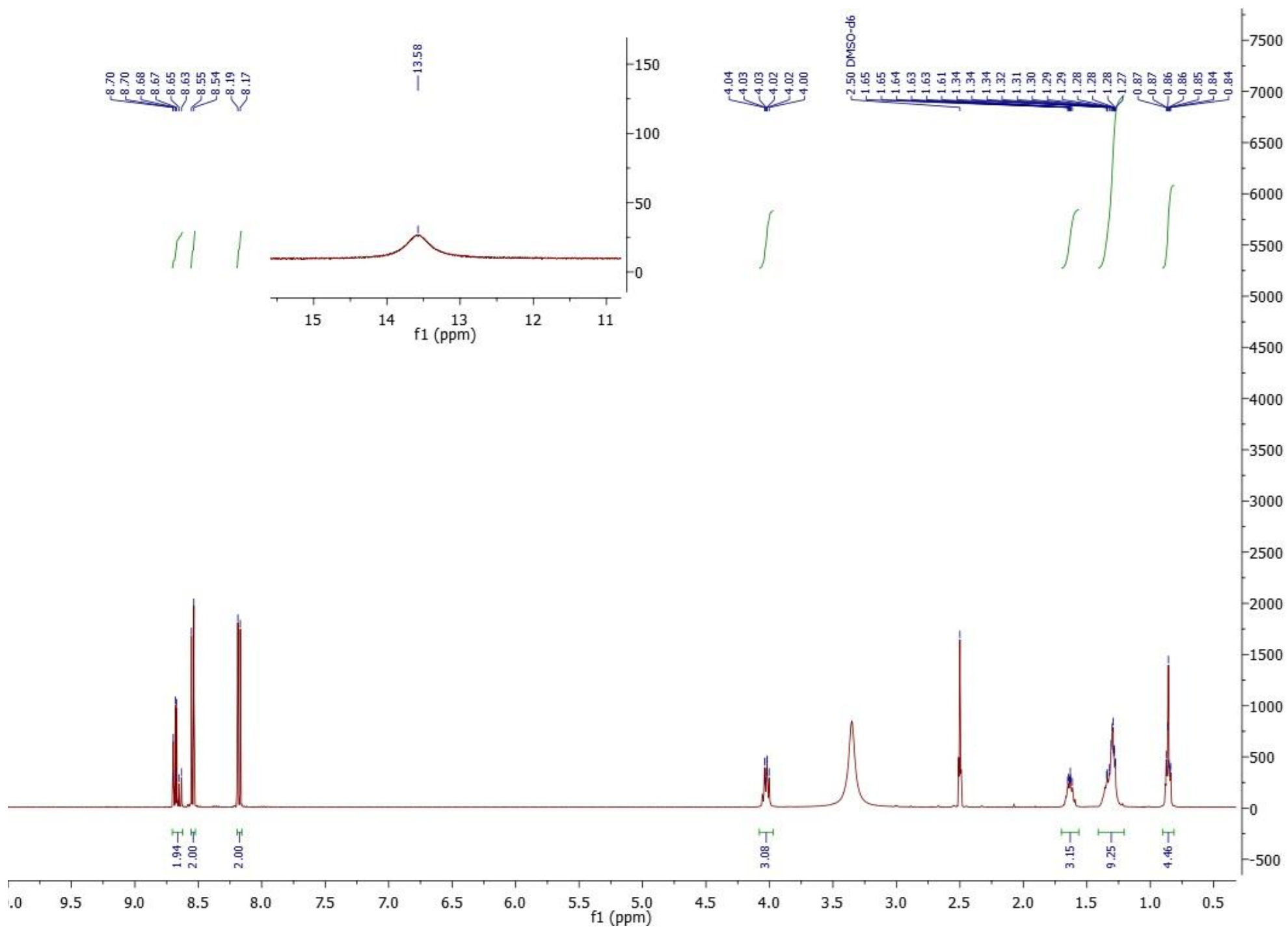

Figure S 4. ^{1}H NMR spectra of **A** in DMSO-d_6.

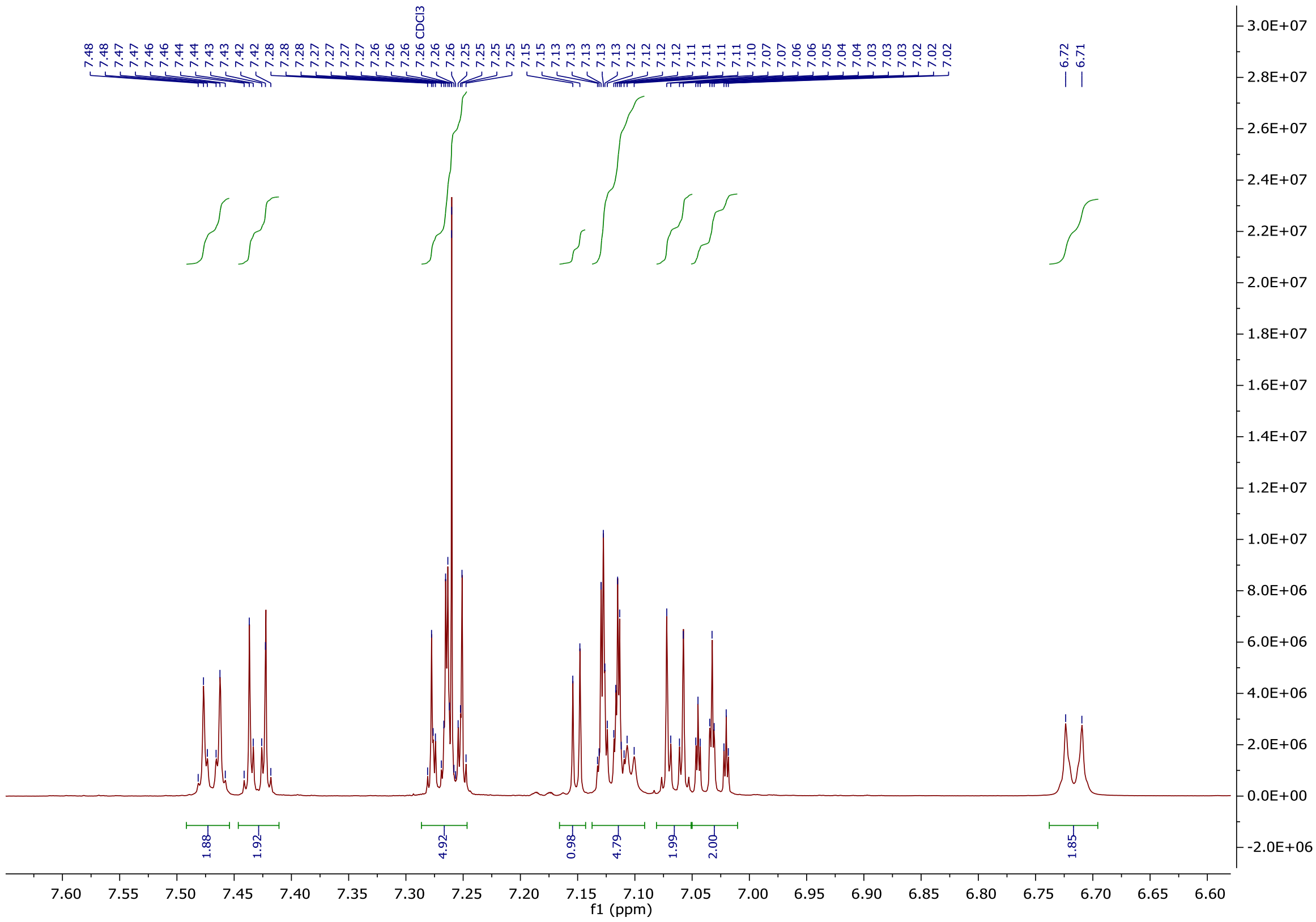

Figure S 5. ^{1}H NMR spectra of **C** in $CDCl_3$.

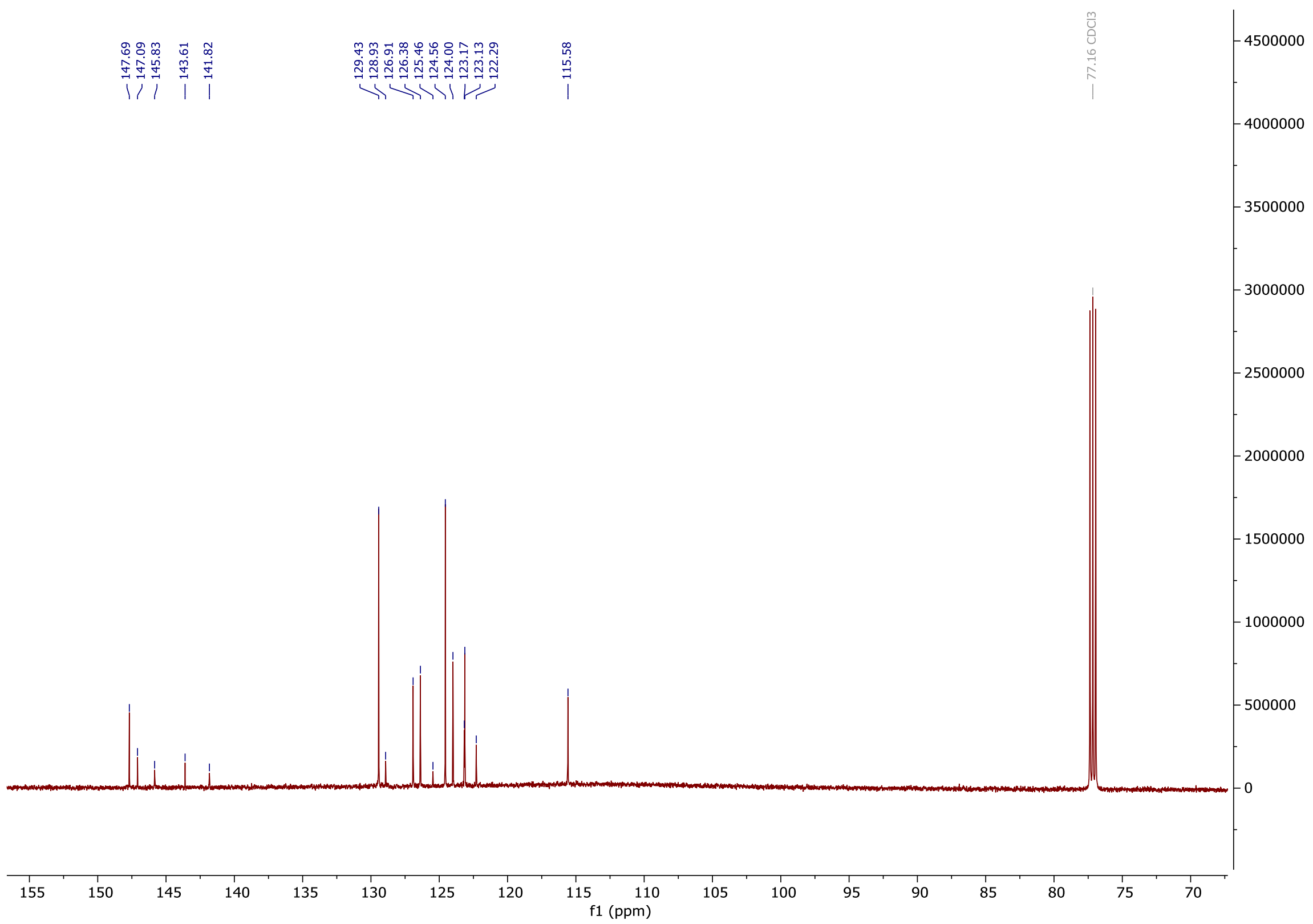

Figure S 6. ^{13}C NMR spectra of **C** in $CDCl_3$.

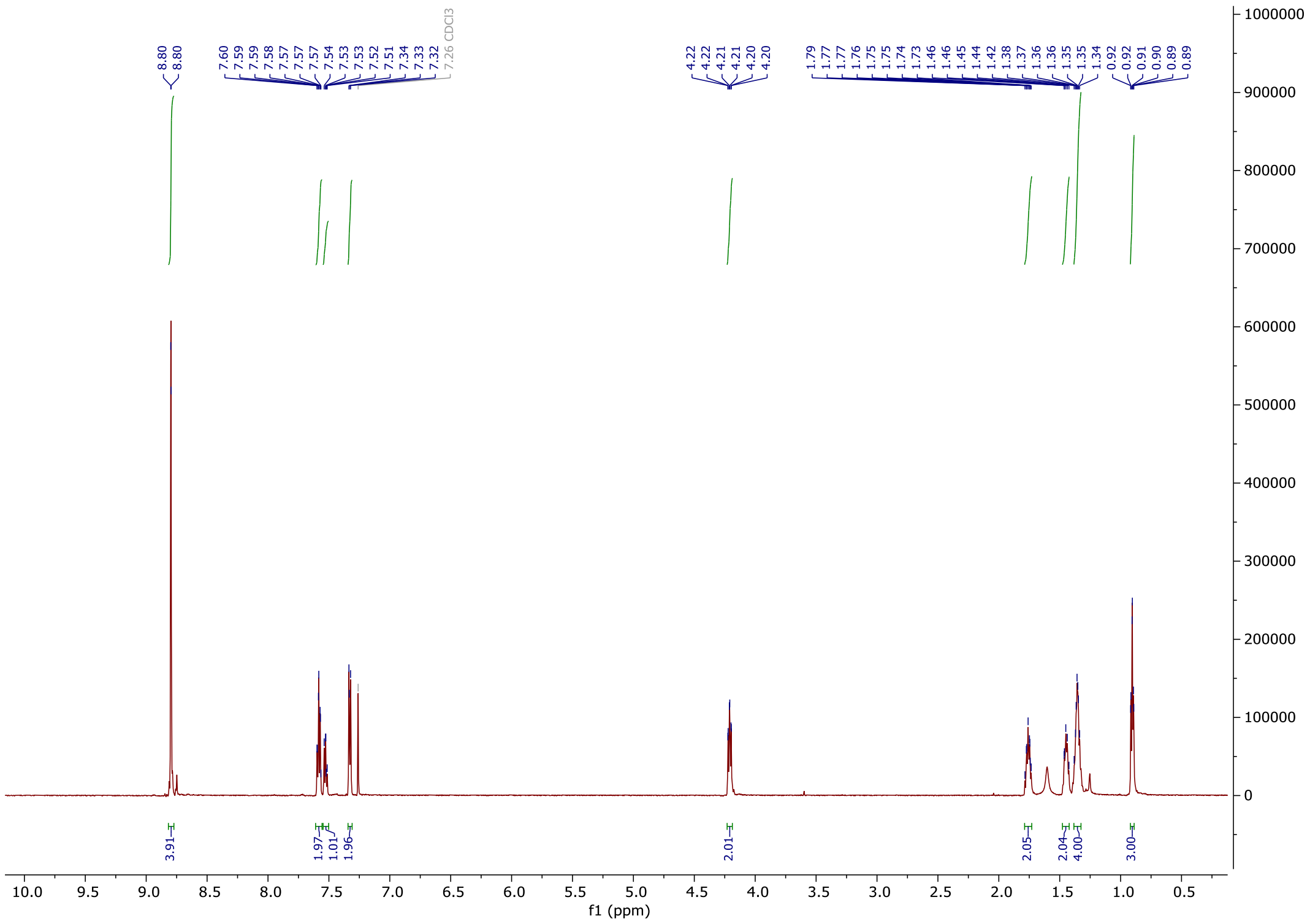

Figure S 7. ^{1}H NMR spectra of **NDI-1** in $CDCl_3$.

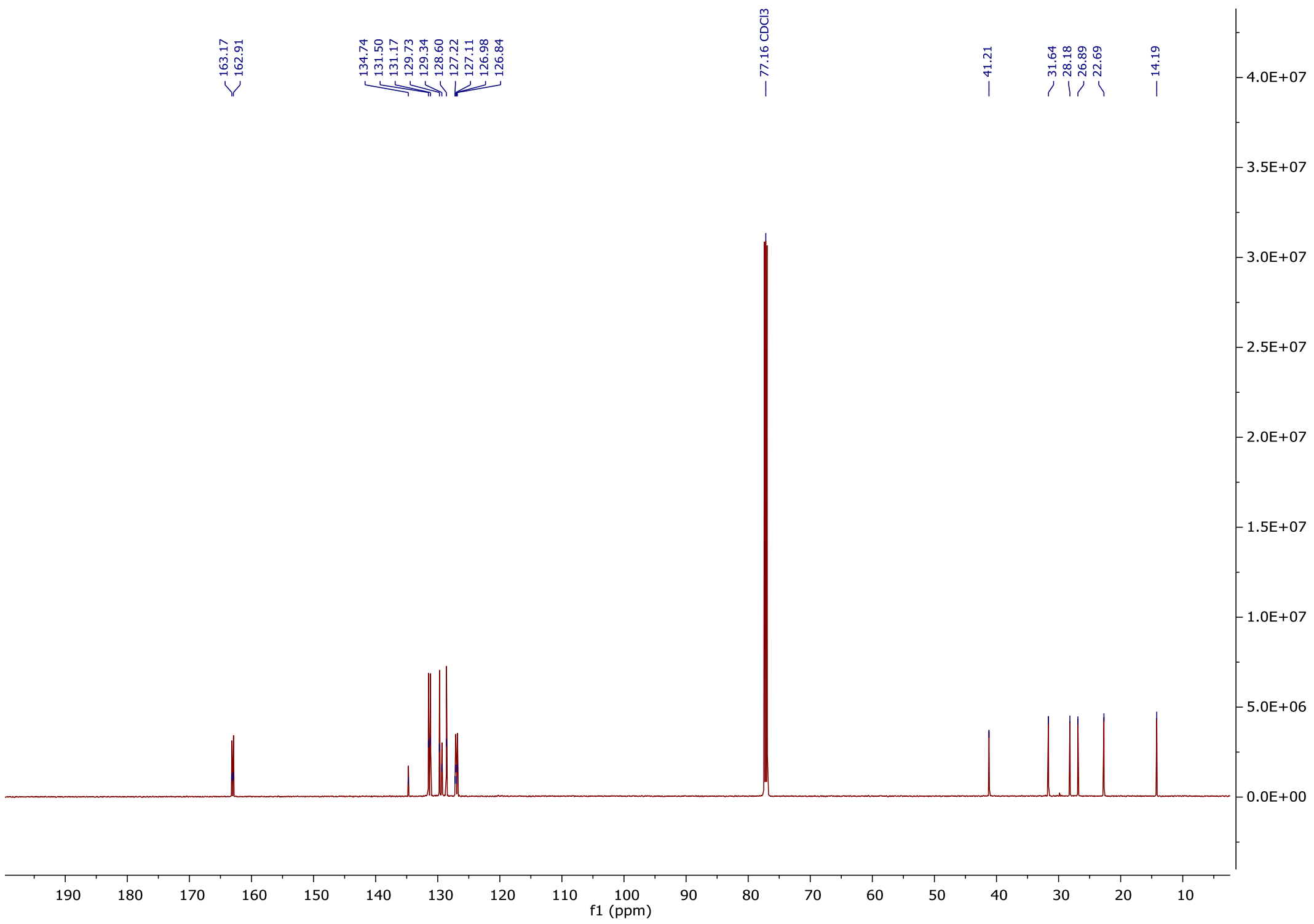

Figure S 8. ^{13}C NMR spectra of **NDI-1** in $CDCl_3$.

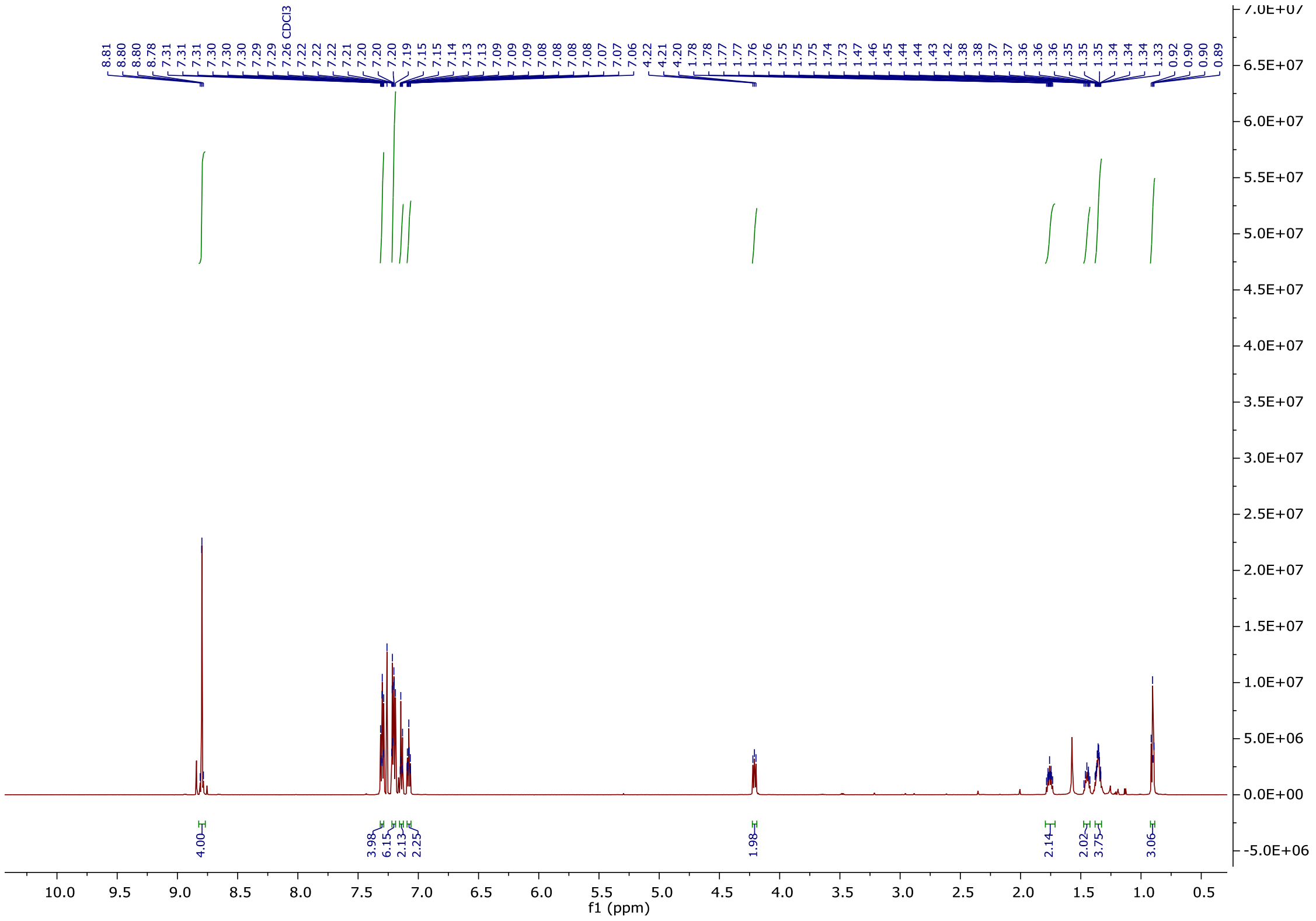

Figure S 9. ^{1}H NMR spectra of **NDI-2** in $CDCl_3$.

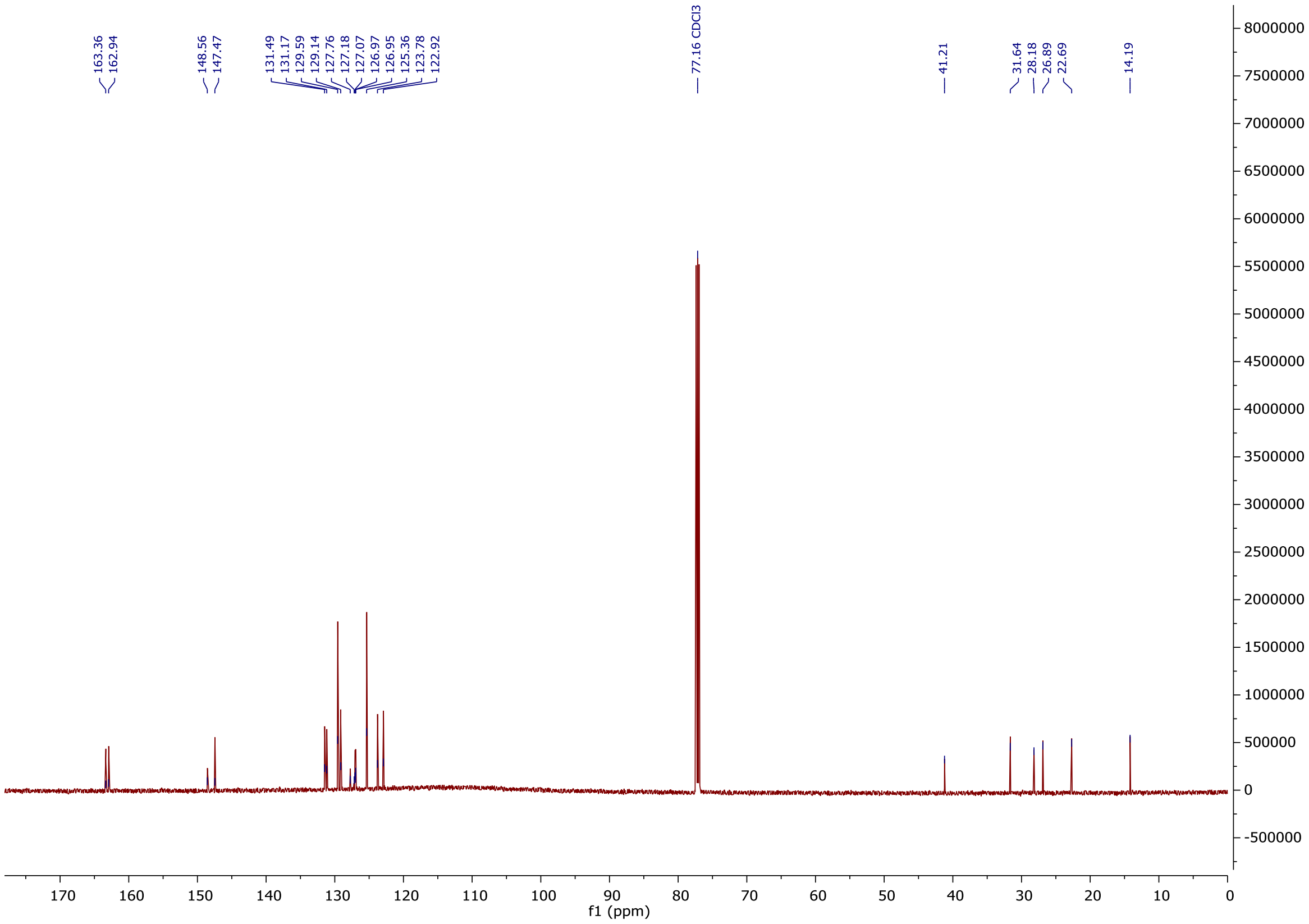

Figure S 10. ^{13}C NMR spectra of **NDI-2** in $CDCl_3$.

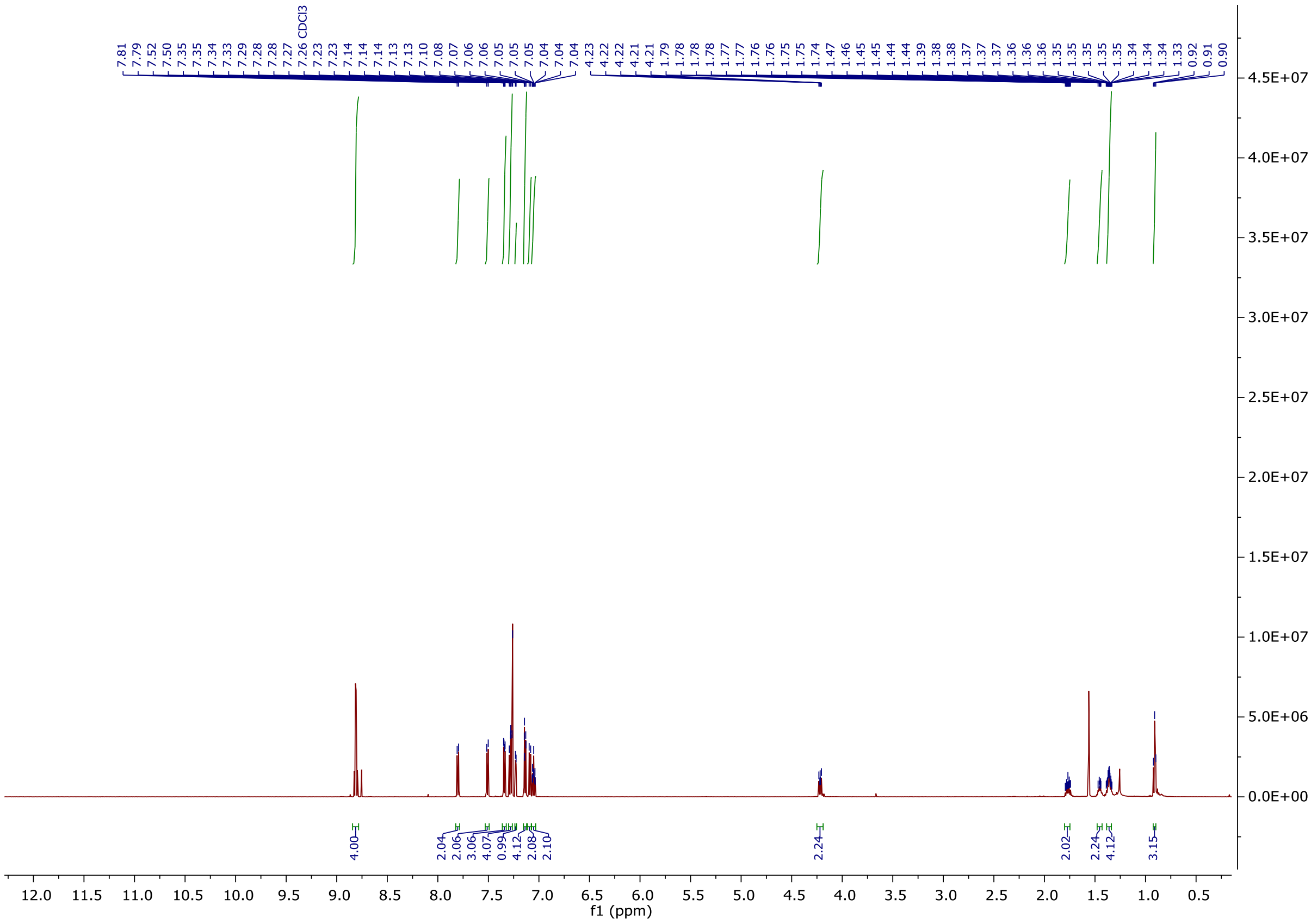

Figure S 11. ^{1}H NMR spectra of **NDI-3** in $CDCl_3$.

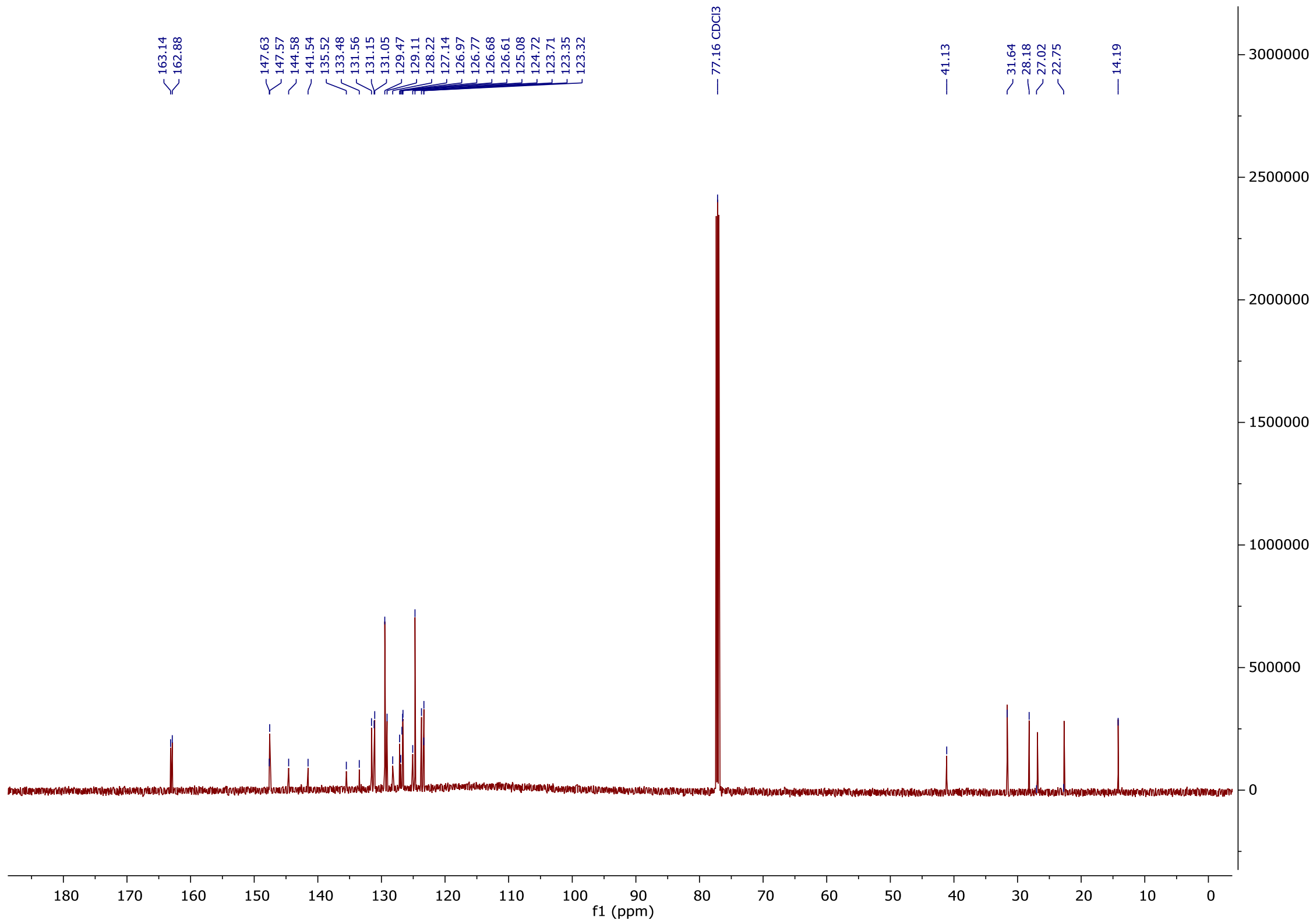

Figure S 12. ^{13}C NMR spectra of **NDI-3** in $CDCl_3$.

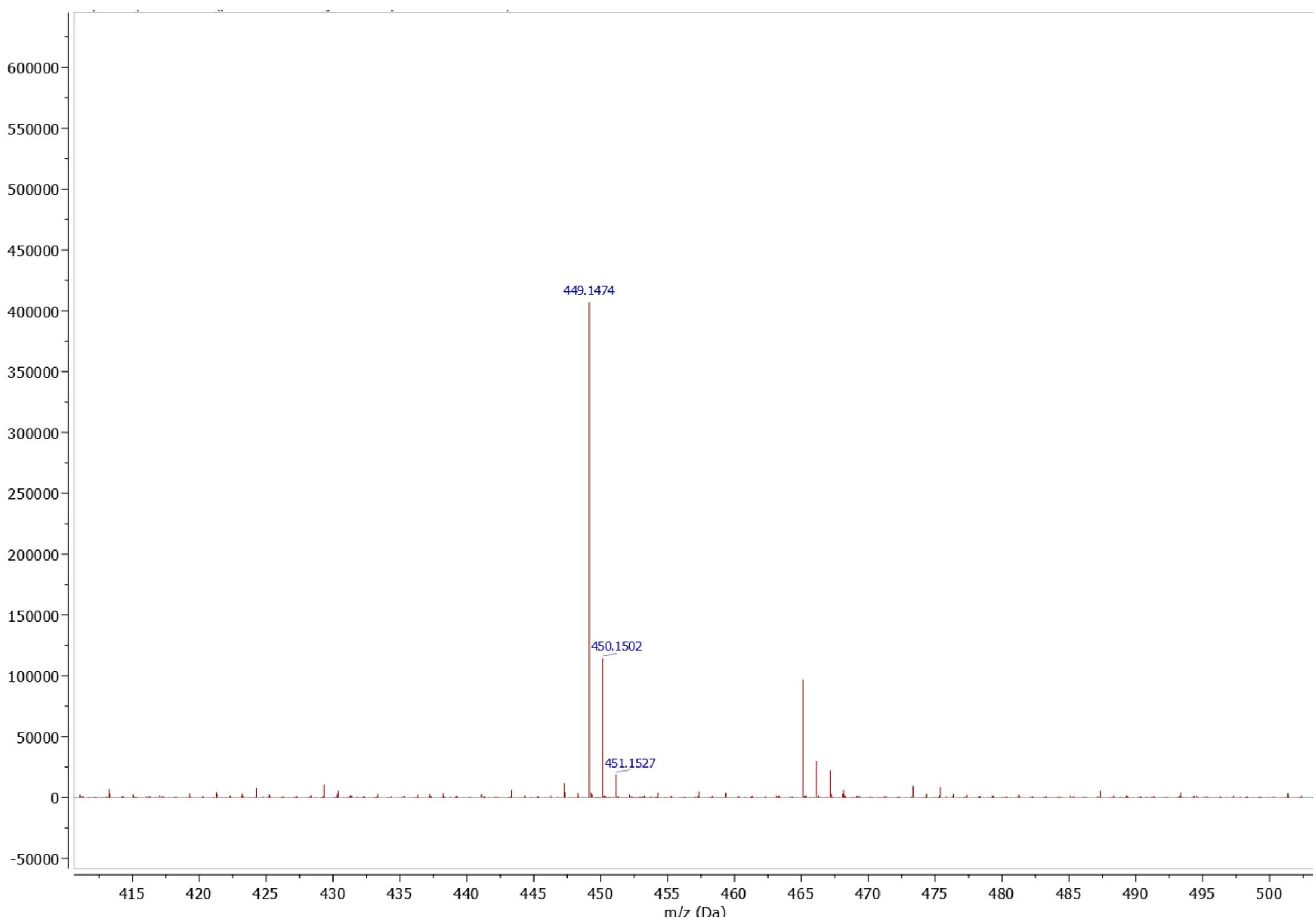

Figure S 13. ESI-MS spectra of compound **NDI-1**.

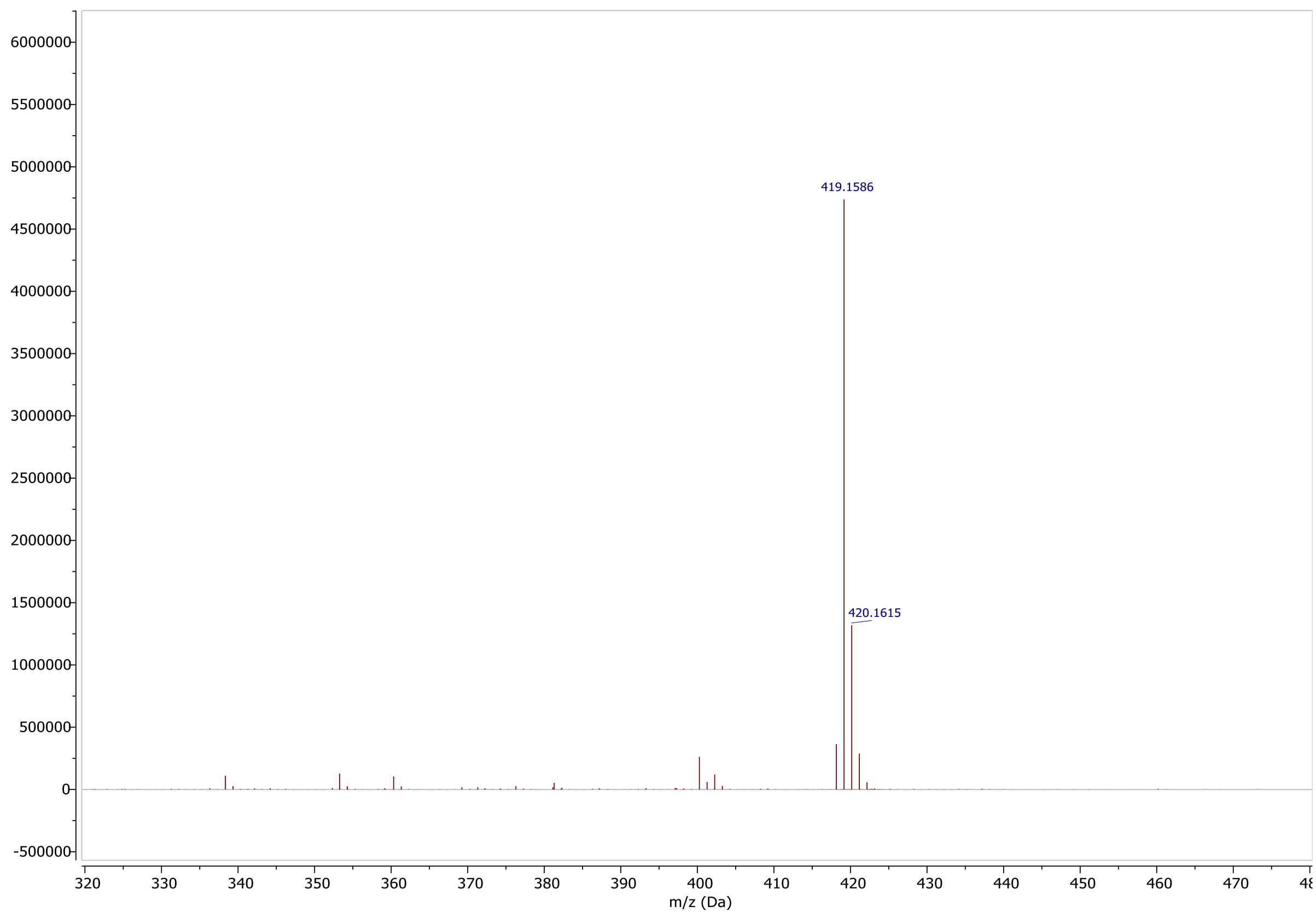

Figure S 14. ESI-MS spectra of compound **C**.

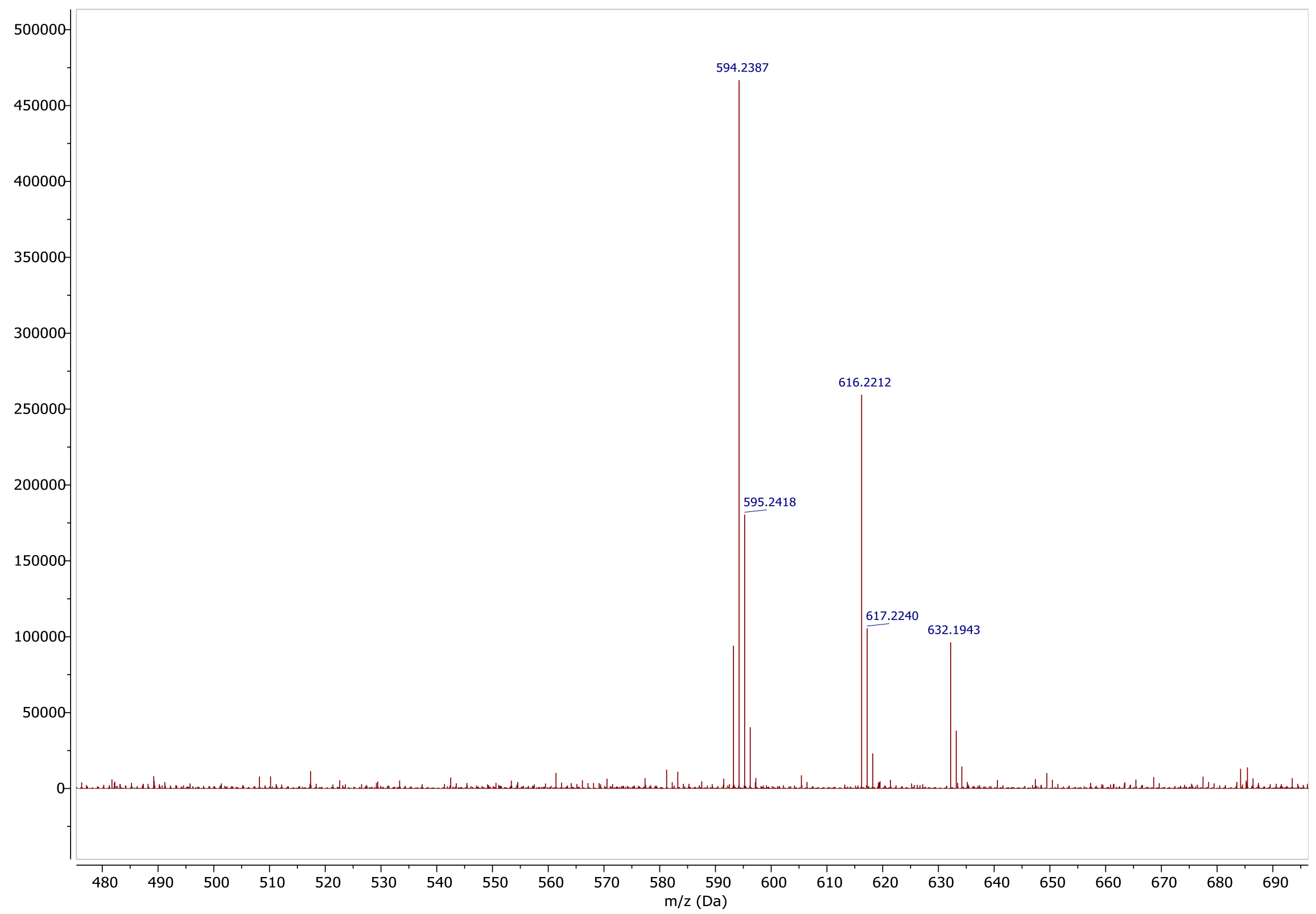

Figure S 15. ESI-MS spectra of compound **NDI-2**.

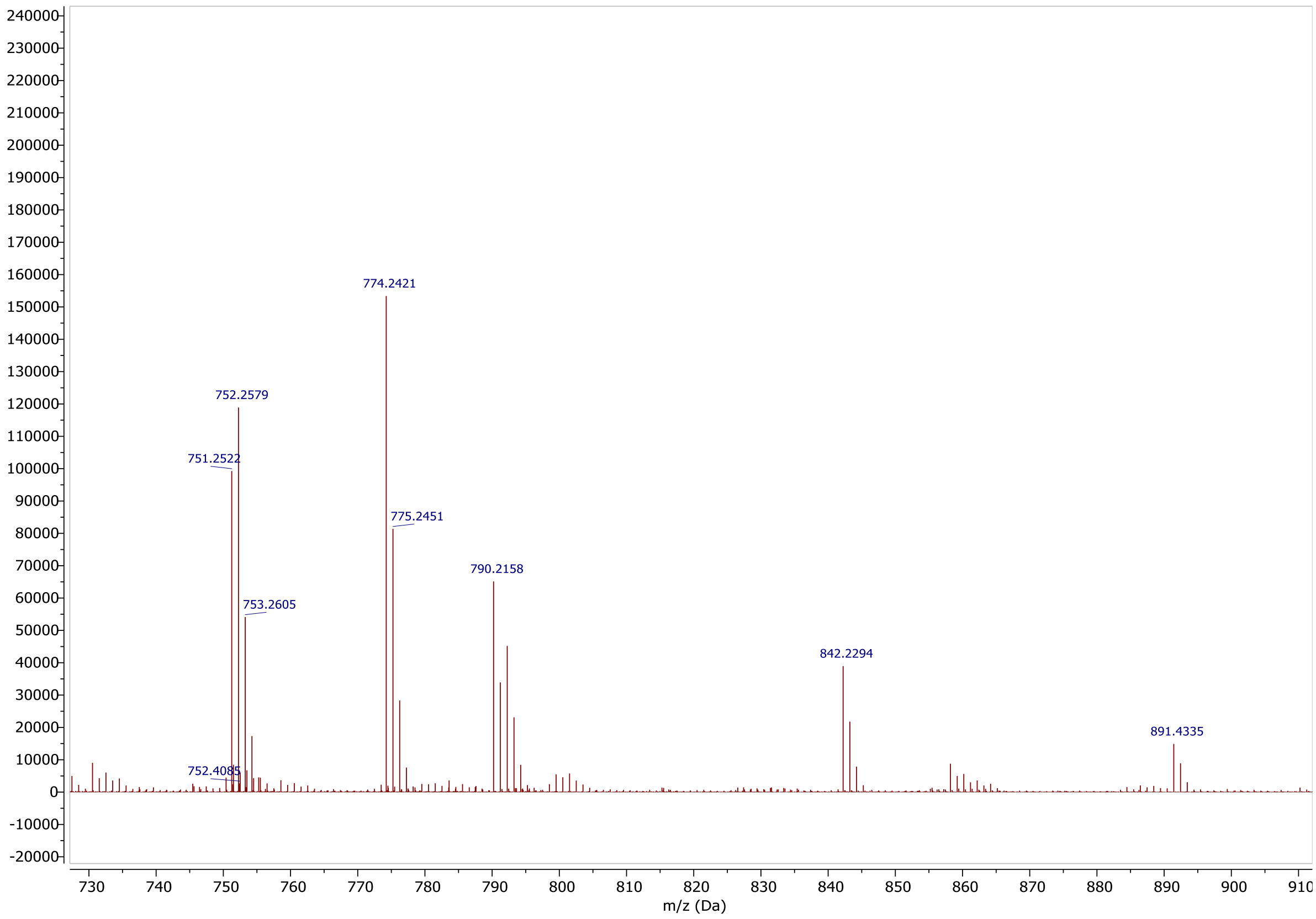

Figure S 16. ESI-MS spectra of compound **NDI-3**.

References:

[1] M. Wałęsa-Chorab, W.G. Skene, Investigation of an electroactive immobilized azomethine for potential electrochromic use, Solar Energy Materials and Solar Cells 200 (2019) 109977. https://doi.org/10.1016/j.solmat.2019.109977.

[2] N. Zhou, K. Prabakaran, B. Lee, S.H. Chang, B. Harutyunyan, P. Guo, M.R. Butler, A. Timalsina, M.J. Bedzyk, M.A. Ratner, S. Vegiraju, S. Yau, C.-G. Wu, R.P.H. Chang, A. Facchetti, M.-C. Chen, T.J. Marks, Metal-Free Tetrathienoacene Sensitizers for High-Performance Dye-Sensitized Solar Cells, J. Am. Chem. Soc. 137 (2015) 4414–4423. https://doi.org/10.1021/ja513254z.
